# Neutrophil extracellular traps and fibrocytes in ST-segment elevation myocardial infarction

**DOI:** 10.1007/s00395-019-0740-3

**Published:** 2019-07-16

**Authors:** Thomas M. Hofbauer, Andreas Mangold, Thomas Scherz, Veronika Seidl, Adelheid Panzenböck, Anna S. Ondracek, Julian Müller, Matthias Schneider, Thomas Binder, Lena Hell, Irene M. Lang

**Affiliations:** 1 Division of Cardiology, Department of Internal Medicine II, Medical University of Vienna, Waehringer Guertel 18-20, 1090 Vienna, Austria; 2 Division of Haematology and Haemostaseology, Department of Internal Medicine I, Medical University of Vienna, Waehringer Guertel 18-20, 1090 Vienna, Austria

**Keywords:** ST-segment elevation myocardial infarction, Neutrophil extracellular traps, Fibrocytes, Thrombosis

## Abstract

Leukocyte-mediated inflammation is central in atherothrombosis and ST-segment elevation myocardial infarction (STEMI). Neutrophil extracellular traps (NETs) have been shown to enhance atherothrombosis and stimulate fibroblast function. We analyzed the effects of NETs on cardiac remodeling after STEMI. We measured double-stranded (ds)DNA and citrullinated histone H3 (citH3) as NET surrogate markers in human culprit site and femoral blood collected during primary percutaneous coronary intervention (*n* = 50). Fibrocytes were characterized in whole blood by flow cytometry, and in culprit site thrombi and myocardium by immunofluorescence. To investigate mechanisms of fibrocyte activation, isolated NETs were used to induce fibrocyte responses in vitro. Enzymatic infarct size was assessed using creatine-phosphokinase isoform MB area under the curve. Left ventricular function was measured by transthoracic echocardiography. NET surrogate markers were increased at the culprit site compared to the femoral site and were positively correlated with infarct size and left ventricular dysfunction at follow-up. In vitro, NETs promoted fibrocyte differentiation from monocytes and induced fibrocyte activation. Highly activated fibrocytes accumulated at the culprit site and in the infarct transition zone. Our data suggest that NETs might be important mediators of fibrotic remodeling after STEMI, possibly by stimulating fibrocytes.

**Electronic supplementary material:**

The online version of this article (10.1007/s00395-019-0740-3) contains supplementary material, which is available to authorized users.

## Introduction

ST-segment elevation myocardial infarction (STEMI) is among the leading causes of death and morbidity [20]. Mounting evidence implicates the innate immune system to be critically involved in atherothrombosis and STEMI [35, 37, 38]. Neutrophil extracellular traps (NETs) are an effector mechanism of neutrophil granulocytes which, upon stimulation, release DNA, histones and granule proteins to form an intravascular fibril matrix [3] that acts as a scaffold for thrombosis [14]. Recently, we described accumulation of NETs at the culprit site in STEMI, and NET burden was positively correlated with myocardial infarct size [38]. Beyond that, NETs are important for age-related organ fibrosis [39] and induce pulmonary fibrosis by stimulating proliferation and differentiation of fibroblasts [8].

Long-term outcome after STEMI is significantly affected by adverse fibrotic remodeling, with fibroblasts being major determinants [5]. Factors triggering this remodeling process are largely unknown. Fibrocytes are circulating cells with features of both leukocytes and fibroblasts [50] and have recently been implicated to drive fibrotic processes [27, 36, 54]. Fibrocytes were shown to be increased in myocardium of patients with coronary artery disease [34] and were implicated in atherosclerosis and in MI [13, 43]. However, the role of fibrocytes in STEMI, especially at the site of coronary occlusion, remains incompletely understood.

We queried whether NETs exerted a fibrotic stimulus in the setting of STEMI, and whether this stimulus occurred via fibrocytes, leading to adverse cardiac remodeling after STEMI.

## Methods

Detailed information on material is provided in Supplemental Table S1.

### Sample collection from patients and healthy controls

We enrolled patients with STEMI (*n* = 50) undergoing primary percutaneous intervention (pPCI) for a coronary TIMI flow of 0. Patients were included if they met the following criteria: (1) chest pain at the time of coronary angiography; (2) new ST-segment elevations of ≥ 2 mm on at least one chest lead or elevations of ≥ 1 mm on at least one limb lead within 20 min of angiography; and (3) suitable coronary anatomy for thrombus aspiration. All other inclusion and exclusion criteria, and general procedural considerations for use of a thrombectomy device, were applied as previously described [38]. All patients received 250 mg of acetylsalicylic acid and were heparinized at an activated coagulation time of > 300 s (4000–10000 IE). Patients with immunosuppression, acute or chronic infections, or autoimmune diseases were excluded. In brief, in all 50 patients, we collected 10–20 ml of blood in close proximity to the culprit site via a commercially available thrombectomy catheter. Blood drawn from the femoral sheath served as comparator. To account for dilutions, all analyses were normalized to femoral hematocrit. Particulate thrombus material was recovered in 21 of these patients, separated using a 40 µm cell strainer and processed for subsequent histology and immunofluorescent staining. In the remaining patients, no thrombus could be harvested due to its disintegration during thrombus aspiration. In 21 patients, venous whole blood was drawn 72 h after pPCI. Furthermore, venous whole blood from healthy donors (*n* = 21, mean age 51 ± 8, no prior diseases or medication) was drawn. All blood samples were immediately centrifuged at 1000*g* for 10 min, and plasma was stored in aliquots at − 80 °C until further analysis. Histological sections of left ventricular myocardium were harvested from patients who suffered from STEMI, or from patients free from cardiac disease. Schematic representation of sampling and experimental procedures is summarized in Supplemental Fig. S1.

### Measurement of soluble NET surrogate markers

Double-stranded DNA (dsDNA) was measured using the Quant-iT PicoGreen Kit as previously described [38]. In brief, plasma samples or standard were incubated with PicoGreen reagent, a fluorescent dye staining free dsDNA, for 5 min in 96-well microplates. Fluorescence was measured using a Promega GloMax Discover microplate reader (excitation 480 nm, emission 520 nm) and is directly proportional to dsDNA concentration in samples. Values were normalized to the standard curve.

Citrullinated histone H3 (citH3) was measured as previously described [64], with minor modifications. Streptavidin-coated 96-well plates were incubated with anti-histone biotin provided in the Cell Death Detection ELISA PLUS Kit for 2 h. Plates were washed and incubated with 50 µl undiluted plasma or standard per well for 1.5 h. After washing, anti-citrullinated histone H3 was incubated for 1 h. Plates were washed and incubated with horse-radish peroxidase conjugate antibody for 1 h. After washing, enzymatic reaction was started by adding BM Blue POD substrate. Plates were stopped after 20 min using 2 M H_2_SO_4_. Optical density was measured on a Promega GloMax Discover microplate reader (450 nm, reference 620 nm) and normalized to the standard curve.

### Assessment of enzymatic infarct size

Enzymatic infarct size was calculated as the area under the curve of creatine-phosphokinase isoform MB (CK-MB AUC) using the trapezoidal formula [10] and expressed as arbitrary units.

### Measurement of functional extracellular vesicle-associated tissue factor

We measured functional extracellular vesicle-associated tissue factor (EV-TF) activity as previously described [22, 28]. We opted for measuring EV-TF instead of sole TF due to the higher reliability of the assay [24]. In brief, EVs were isolated from citrated platelet poor plasma by centrifugation at 18,000*g*, 20 min at 4 °C, washed twice with HBSA (137 mmol/l sodium chloride, 5.38 mmol/l potassium chloride, 5.55 mmol/l glucose, 10 mmol/l HEPES, 0.1% bovine serum albumin, pH 7.5) buffer and resuspended in 200 µl HBSA. EV suspension was then incubated with an antibody for human TF (hTF1, 1 µl) or control antibody (mouse IgG, 1 μl) for 15 min at 21 °C. 50 μl aliquots were then added to 96-well plates in duplicates. 50 μl of HBSA containing 10 nmol/l factor VIIa, 300 nmol/l factor X (FX) and 10 mmol/l calcium chloride was added to each sample and incubated for 2 h at 37 °C. FXa generation was stopped by the addition of 25 μl of disodium ethylenediaminetetraacetate (EDTA) buffer and 25 μl of the chromogenic substrate Pefachrome FXa 8595 (4 mmol/l) was added and incubated at 37 °C for 15 min. Absorbance at 405 nm was measured using a Multiscan Spectrum microplate reader (Thermo Scientific). TF-dependent FXa generation (pg/ml), which represents EV-associated TF activity, was determined by subtracting the amount of FXa generated in the presence of hTF1 from the amount of FXa generated in the presence of the control antibody.

### Isolation of NETs

NETs were isolated as previously described [46]. 30 ml of heparinized whole blood of healthy donors was layered on top of 15 ml Lymphocyte Separation Medium and centrifuged at 800*g* for 30 min at 21 °C with brakes off. Plasma and peripheral blood mononuclear cell (PBMC) layers were discarded. Then, 20 ml 6% dextran and 20 ml sterile phosphate-buffered saline (PBS) were gently mixed with the remaining cell suspension containing erythrocytes and neutrophils. After incubation for 30 min at 21 °C, the supernatant containing neutrophils was collected and washed with sterile PBS. Remaining erythrocytes were lysed using red cell lysis buffer (154 mmol/l ammonium chloride, 10 mmol/l potassium hydrogen carbonate, 0.1 mmol/l EDTA). Neutrophil purity was assessed using a XN-350 Hematology Analyzer (Sysmex) and was usually above 95%. 5 × 10^6^/ml neutrophils resuspended in RPMI + 3% fetal calf serum (FCS) were seeded into 6-well cell culture plates and were stimulated with 500 nmol/l phorbol myristate acetate for 4 h at 37 °C. Supernatant was discarded, and ice-cold PBS was used to detach generated NETs from the bottom of the wells. After centrifugation at 450 g for 10 min at 4 °C, cell-free, NET-rich supernatant was collected and stored at − 80 °C. Concentration of NETs was measured using PicoGreen reagent as described above.

### Isolation of peripheral blood mononuclear cells

15 ml of heparinized blood from healthy donors and STEMI patients was layered onto 15 ml of lymphocyte separation medium in 50 ml tubes and centrifuged for 15 min, 800*g* at 21 °C with brakes off. The PBMC layer was collected and washed with PBS + 10% FCS. Cells were then incubated with red cell lysis buffer to remove remaining erythrocytes and washed with PBS.

### In vitro stimulation of fibrocytes with NETs

To assess the impact of NET exposure on fibrocyte differentiation, 1 × 10^6^/ml isolated PBMCs were seeded into 8-well chamber slides and stimulated with 500 ng/ml isolated NETs, NETs with 40 IE/ml DNase 1, NETs with 20 µg/µl anti-TLR-4 blocking antibody, NETs with DNase and anti-TLR-4 blocking antibody, or vehicle control. Medium was changed after 24 h, and cells were again stimulated as described above. After another 48 h of stimulation, cells were washed with medium, fixed in 4% paraformaldehyde for 1 h and stained with hematoxylin for 10 min. Slides were then rinsed with distilled water and mounted using glass coverslips. Fibrocytes were identified as spindle-shaped cells in twenty random fields (20×) of each experimental condition. Images were acquired using an Olympus DP72 microscope by an observer blinded to experimental conditions. Cells were then counted using ImageJ software (Version 1.51j8).

To test NET-induced fibrocyte activation, PBMCs (1 × 10^6^/ml) were resuspended in RPMI containing 10% FCS, l-glutamine, 1% MEM non-essential amino acids, 50 µg/ml penicillin, 50 µg/ml streptomycin, 50 µg/ml gentamycin and 2.5 µg/ml fungizone, seeded into 24-well cell culture plates and incubated for 72 h at 37 °C, which leads to spontaneous differentiation of a fraction of monocytes into fibrocytes [50]. Cells were incubated with Protein Transport Inhibitor (BD GolgiPlug^®^) for 5 min and subsequently stimulated with 500 ng/ml of isolated NETs, NETs + 40 IE/ml DNase 1 (Dornase alfa, Pulmozyme^®^) or vehicle control for 6 h. Medium was aspirated, and cells were detached using trypsin–EDTA. After three washing steps using PBS, fibrocytes were characterized using flow cytometry as described below.

### Flow cytometry

Fibrocytes from in vitro stimulation experiments were incubated with primary fluorescent antibodies against collagen-I, CD34, CD45 and primary unconjugated goat IgG BMPRII for 15 min. After washing with PBS, a secondary fluorescent anti-goat IgG antibody was added for 15 min. In addition, intracellular IL-6 was measured. After incubation with extracellular primary antibodies, cells were incubated with FIX & PERM fixation medium for 15 min. After washing, cells were incubated with FIX & PERM permeabilization medium and intracellular IL-6 antibody for 15 min.

Whole blood samples collected in EDTA-containing tubes were incubated with primary fluorescent antibodies against collagen-I, CD34, CD45, CD11b and primary unconjugated goat IgG BMPRII for 15 min. Red blood cells were lysed using BD FACS lysis solution. After washing with PBS, a secondary fluorescent anti-goat IgG antibody was added for 15 min. Cells were then washed and resuspended in PBS for analysis.

Cells were analyzed with a BD FACSCanto II and FACSDiva Software (BD Biosciences). Cells were separated from debris by adjusting forward and side scatter. Leukocytes were identified based on CD45 expression. Frequency of circulating fibrocytes, defined as CD45^+^CD34^+^collagen-I^+^ cells [50], was computed as fibrocytes/10^6^ CD45^+^ cells. Surface marker expression is given as mean fluorescence intensity (MFI). An example of gating from a representative whole blood patient sample is shown in Supplemental Fig. S2. MFI values and statistics are provided in Supplemental Tables 2, 3, and 4.

### Immunofluorescence

Upon collection, tissue specimens were immediately immersed in 7.5% formalin for 24 h, embedded in paraffin and then cut into 3 µm sections for immunofluorescence staining. For staining of fibrocytes, samples were stained using primary antibodies against CD34, CD45 and collagen-I at 4 °C overnight. As secondary antibodies, DyLight 550, DyLight 755 and DyLight 650 were used at room temperature for 1 h. For staining of NETs, culprit site thrombi were incubated using a primary antibody against DNA-Histone H1 at 4 °C overnight. As secondary antibody, DyLight 755 was used at room temperature for 1 h. Nuclei were stained using DAPI. Images were acquired with an Axio Observer Z1 fluorescence microscope. Analysis of images was performed using TissueQuest software (TissueGnostics, version 4.01.0128).

### Trichrome staining

Modified trichrome stains were performed as previously described [17]. Image acquisition and analysis were performed using an Olympus DP72 microscope.

### Echocardiographic assessment of left ventricular function

In a subset of 33 patients, transthoracic echocardiography was performed 3 [2, 4] days after STEMI. Echocardiography at long-term follow-up (24 ± 8 months) was performed in 24 patients. Left ventricular ejection fraction (LVEF) was measured using biplane modified Simpson’s method. Myocardial contractility was assessed and scored for each segment as described [26]: (1) normal or hyperkinesia; (2) hypokinesia; (3) akinesia; (4) dyskinesia. Wall Motion Score Index (WMSI) was computed as the sum of the score of each segment divided by the total number of segments.

### Statistical analysis

Distribution of data was analyzed using the Kolmogorov–Smirnov test and histograms (data not shown). Normally distributed numerical data are presented as mean ± standard deviation (SD); otherwise, median and interquartile range (IQR) are provided. To compare paired and normally distributed data, paired Student *t* test was used; otherwise, Wilcoxon signed-rank test was computed. For unpaired comparisons, unpaired Student’s *t* test was employed in case of normally distributed data; otherwise, Mann–Whitney *U* test was used. For comparison of three groups, one-way analysis of variance with Dunn’s multiple post hoc comparison was employed. Correlations between parameters were assessed using Spearman’s rank correlation. To correct for multiple testing, the Bonferroni–Holm method was used. Statistical analyses were performed using IBM SPSS 21.0. Figures were generated using GraphPad Prism 5. Data are depicted as scatter plots; lines in graphs indicate mean ± standard error of the mean, in case of normally distributed data; otherwise, median and IQR are given.

## Results

### Patient characteristics

Characteristics of patients with STEMI and TIMI flow of 0 at pPCI are shown in Table 1. Two patients (4%) died in hospital.

**Table 1:** Patient characteristics

Patient characteristics (*n* = 50)	
Age, years ± SD	61 ± 12
Male sex, *n* (%)	39 (78)
BMI > 25 kg/m^2^, *n* (%)	34 (68)
BMI > 30 kg/m^2^, *n* (%)	9 (18)
Diabetes, *n* (%)	9 (18)
History of hypertension, *n* (%)	37 (74)
Dyslipidemia, *n* (%)	33 (66)
Ever smoker, *n* (%)	33 (66)
Family history of CAD, *n* (%)	24 (48)
Previous MI, *n* (%)	8 (16)
TIMI flow 0 prior to pPCI	50 (100)
Culprit lesion, *n* (%)	
LAD	25 (50)
CX	8 (16)
RCA	17 (34)
CAD, *n* (%)	
1VD	23 (46)
2VD	13 (26)
3VD	14 (28)
Medication prior to admission, *n* (%)	
Any medication prior to admission	23 (46)
Statin prior to admission	7 (14)
Anti-hypertensive prior to admission	12 (24)
ACE inhibitor prior to admission	7 (14)
ARB prior to admission	3 (6)
Spironolactone prior to admission	0 (0)
ASA prior to admission	19 (38)
CRP, nmol/l (< 4.8)	3.62 [1.90–7.81]
CK-MB, maximum, U/l (< 24)	213 [112–508]
CK-MB AUC	8533 [4049–15570]
TnT at admission, µg/l (0–0.03)	0.05 [0.02–0.11]
Creatinine, µmol/l (50–100)	86.16 ± 29.74
Cholesterol, mmol/l (< 5.2)	4.99 ± 0.85
LDL, mmol/l (< 4.1)	2.74 ± 0.83
HDL, mmol/l (> 1.5)	1.22 ± 0.34
Triglycerides, mmol/l (< 1.7)	1.61 ± 1.12
Time delay, min	194 [146–415]
Culprit site total leukocytes at admission, G/L (< 10)	12.32 [8.58–17.83]
Culprit site neutrophils at admission (%)	79 [73–84]
Culprit site monocytes at admission (%)	5 [4–6]
LVEF after STEMI (< 40%)	49.1 ± 9.4
Wall Motion Score Index at follow-up	1.25 [1.00–1.67]

Data are presented as mean ± standard deviation (SD), median [interquartile range, IQR] or number (percent) of patients. Reference values are provided in parentheses. Time delay refers to the minutes between onset of symptoms and reperfusion

*ACE*-*I* angiotensin converting enzyme inhibitor, *ARB* angiotensin receptor blocker, *ASA* acetylsalicylic acid*, BMI* body mass index, *CAD* coronary artery disease, *CK*-*MB AUC* area under the curve of creatine-phosphokinase isoform MB, *CRP* C-reactive protein, *CX* circumflex artery, *HD*L high-density lipoprotein, *LAD* left anterior descending artery, *LDL* low-density lipoprotein, *MI* myocardial infarction, *LVEF* left ventricular ejection fraction, *pPCI* primary percutaneous coronary intervention, *RCA* right coronary artery, *STEMI* ST-segment elevation myocardial infarction, *TnT* troponin T, *VD* vessel disease

### NETs accumulate at the culprit site and correlate with infarct size

**Fig. 1:**
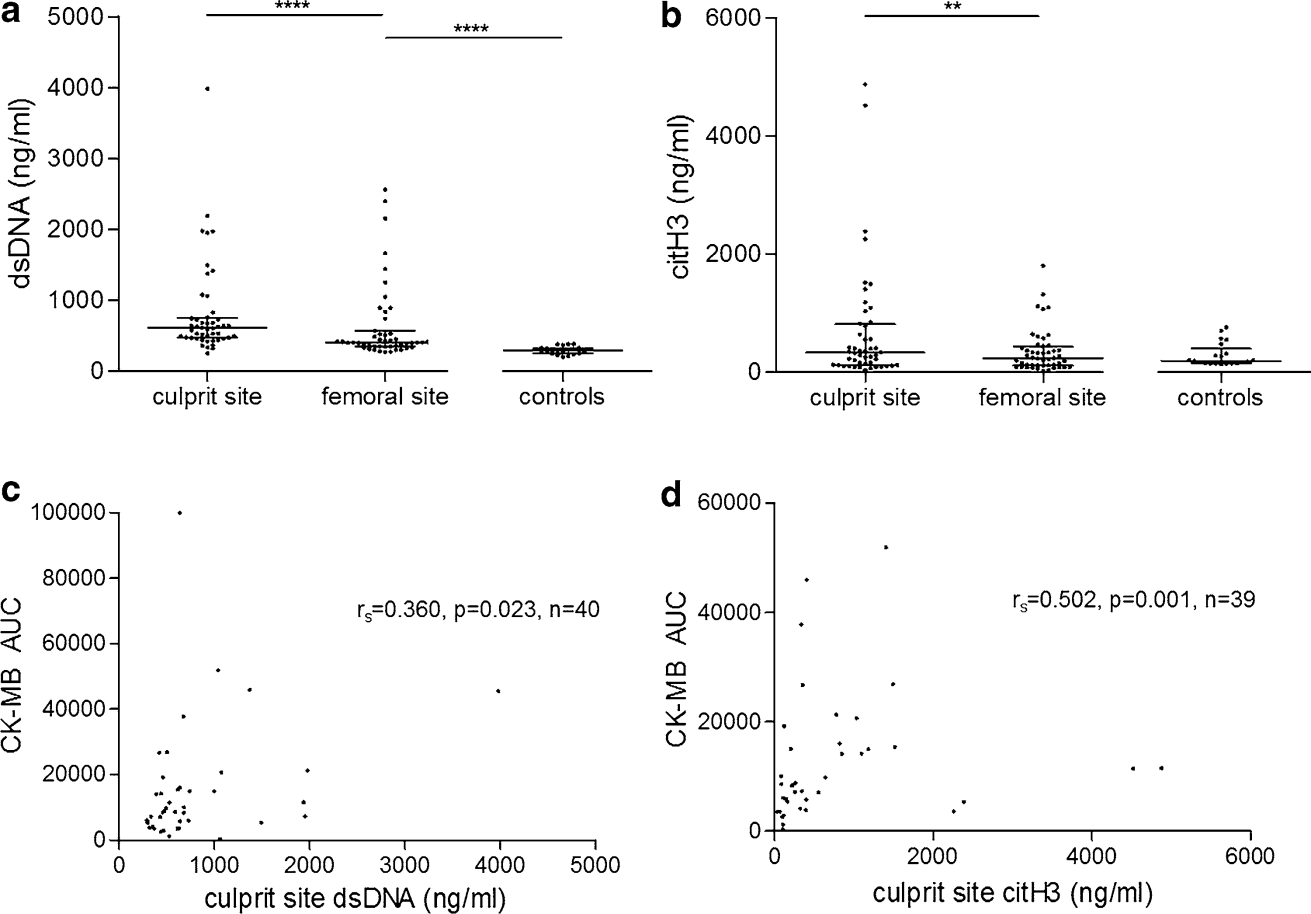
Neutrophil extracellular trap (NET) markers in patients with ST-segment elevation myocardial infarction and healthy controls, and correlation with enzymatic infarct size. **a** Double-stranded (ds)DNA was measured using PicoGreen. **b** Citrullinated histone H3 (citH3) was measured using ELISA. Data are provided as ng/ml. Correlations of NET surrogate markers **c** double-stranded (ds)DNA and **d** citrullinated histone H3 (citH3) at the culprit site with enzymatic infarct size. Infarct size is expressed as creatine-phosphokinase isoform MB area under the curve (CK-MB AUC, given as arbitrary units). For STEMI patients, *n* = 48; for healthy controls, *n* = 21. Lines in **a** and **b** indicate median and interquartile range. Significance was determined by paired Wilcoxon signed-rank and unpaired Mann–Whitney *U* test. Correlation coefficients were calculated by Spearman’s rank correlation. **p < 0.01, ****p < 0.0001

In a first series of experiments, we measured concentrations of dsDNA and citH3 as surrogate markers of NET burden. Consistent with our previous findings [38], dsDNA was significantly increased at the culprit site compared with femoral site dsDNA concentration (*n* = 48, culprit site dsDNA 529 [429–740] vs. femoral dsDNA 404 [349–563] ng/ml, *p* < 0.0001, Fig. 1a). Furthermore, the specific NET marker citH3 was also highly increased at the culprit site (*n* = 48, culprit site 332 [123–810] vs. femoral 235 [113–434] ng/ml, *p* < 0.01, Fig. 1b). Healthy controls displayed lower dsDNA concentrations compared with femoral site concentrations of STEMI patients (controls *n* = 21, 291 [252–327] ng/ml, *p* < 0.0001, Fig. 1a), whereas citH3 levels did not differ significantly (controls *n* = 21, 192 [150–399] ng/ml, *p* = ns, Fig. 1b). No correlations were found between symptom to balloon time and NET burden (data not shown). Both dsDNA (Fig. 1c) and citH3 (Fig. 1d) in culprit site plasma were positively correlated with enzymatic infarct size. No differences in NET burden between culprit sites were observed, neither of soluble NET markers, nor of thrombus NET burden as measured by immunohistochemistry (Supplemental Fig. S4a–c). Similarly, multi-vessel disease was not associated with increased NET burden (Supplemental Fig. S4d–f).

Tissue factor (TF) is important for the primary activation of the coagulation protease cascade and is thus paramount for hemostasis and thrombosis [9, 19]. Recently, TF was shown to be expressed and functionally active on NETs at the culprit site [60]. We found activity of circulating EV-TF to be increased at the culprit site compared to femoral control (*n* = 46, culprit site 0.12 [0.01–0.83] vs. femoral 0.00 [0.00–0.08] pg/ml, *p* < 0.001, Supplemental Fig. S5a). At 72 h after pPCI, EV-TF activity was not different compared with femoral site values (72 h *n* = 21, 0.02 [0.00–0.16] pg/ml, *p* = ns vs. femoral). EV-TF activity at the culprit site was positively correlated with dsDNA (*n* = 46, *r*_s_ = 0.316, *p* < 0.05, Supplemental Fig. S5b), but not with citH3 (*n* = 47, *r*_s_ = 0.275, *p* = ns, Supplemental Fig. S5c).

### NETs induce differentiation of fibrocytes from monocytes in vitro and lead to fibrocyte activation

**Fig. 2:**
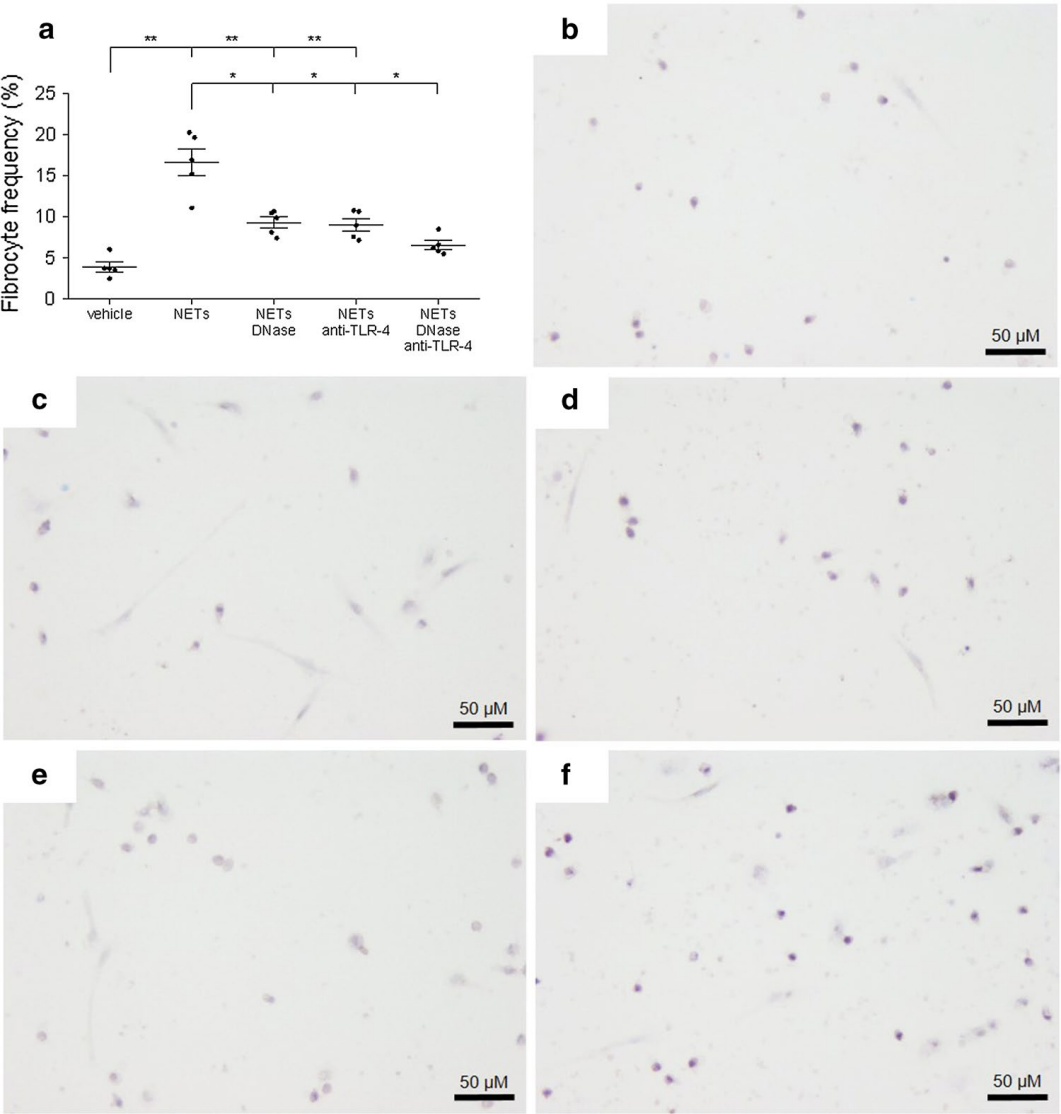
Differentiation of monocytes to fibrocytes by neutrophil extracellular traps (NETs) in vitro. Isolated peripheral blood mononuclear cells containing monocytes were stimulated with vehicle control, 500 ng/ml isolated NETs, NETs plus DNase or NETs plus anti-TLR-4 blocking antibody for 72 h (n = 5). Representative hematoxylin stainings for **b** vehicle control; **c** NETs; **d** NETs plus DNase; **e** NETs plus anti-TLR-4 blocking antibody; and **f** NETs plus DNase + anti-TLR-4 blocking antibody are shown. Data in **a** are given as mean ± standard error of the mean. Significance was determined by one-way analysis of variance with Dunn’s multiple post hoc comparison. *p < 0.05, **p < 0.01

Monocytes have been shown to be precursors of fibrocytes [43]. Upon incubation of cells with isolated NETs, we observed an increased differentiation of monocytes into fibrocytes after 72 h (Fig. 2a). Representative examples for each condition are shown in Fig. 2b–f. Addition of DNase diminished the effect of NETs on fibrocyte differentiation. Addressing the question of mechanisms by which NETs induce monocyte differentiation towards fibrocytes, we employed a TLR-4 blocking antibody and observed that differentiation was blunted, similar to the effect of DNase. Addition of both DNase and TLR-4 blocking antibody exerted no additional inhibitory effect. Monocytes derived from STEMI patients displayed similar levels of fibrocyte differentiation compared to healthy controls (Supplemental Fig. S6).

**Fig. 3:**
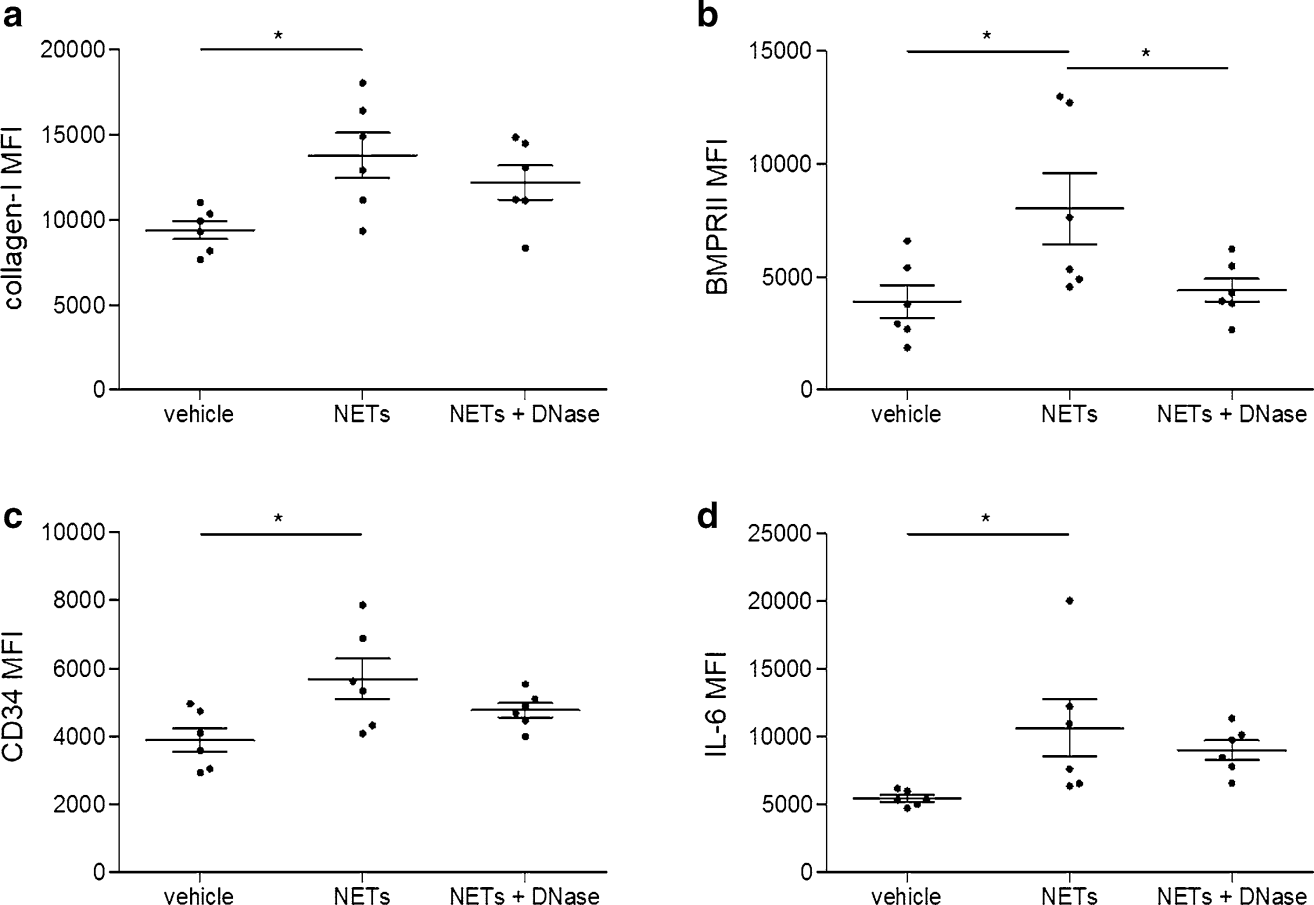
Fibrocyte activation by neutrophil extracellular traps (NETs) in vitro. Fibrocytes were stimulated with vehicle control, 500 ng/ml isolated NETs from healthy donors or NETs plus DNase for 6 h (*n* = 6). Cells were characterized using flow cytometry for their surface expression of **a** collagen-I; **b** bone morphogenic protein receptor II (BMPRII); **c** CD34; and for their intracellular expression of **d** interleukin (IL)-6. Data are provided as mean ± standard error of the mean. Significance was determined by one-way analysis of variance with Dunn’s multiple post hoc comparison. *p < 0.05

In a next step, we stimulated cultivated fibrocytes with isolated NETs in vitro for 6 h. NETs induced an upregulation of collagen-I (Fig. 3a), BMPRII (Fig. 3b) and CD34 (Fig. 3c) compared to control. Furthermore, NETs stimulated the production of IL-6 by fibrocytes (Fig. 3d). Addition of DNase abrogated the increase of BMPRII (Fig. 3b) but had no significant impact on other markers.

### Fibrocytes accumulate at the culprit site and coronary thrombus in STEMI

**Fig. 4:**
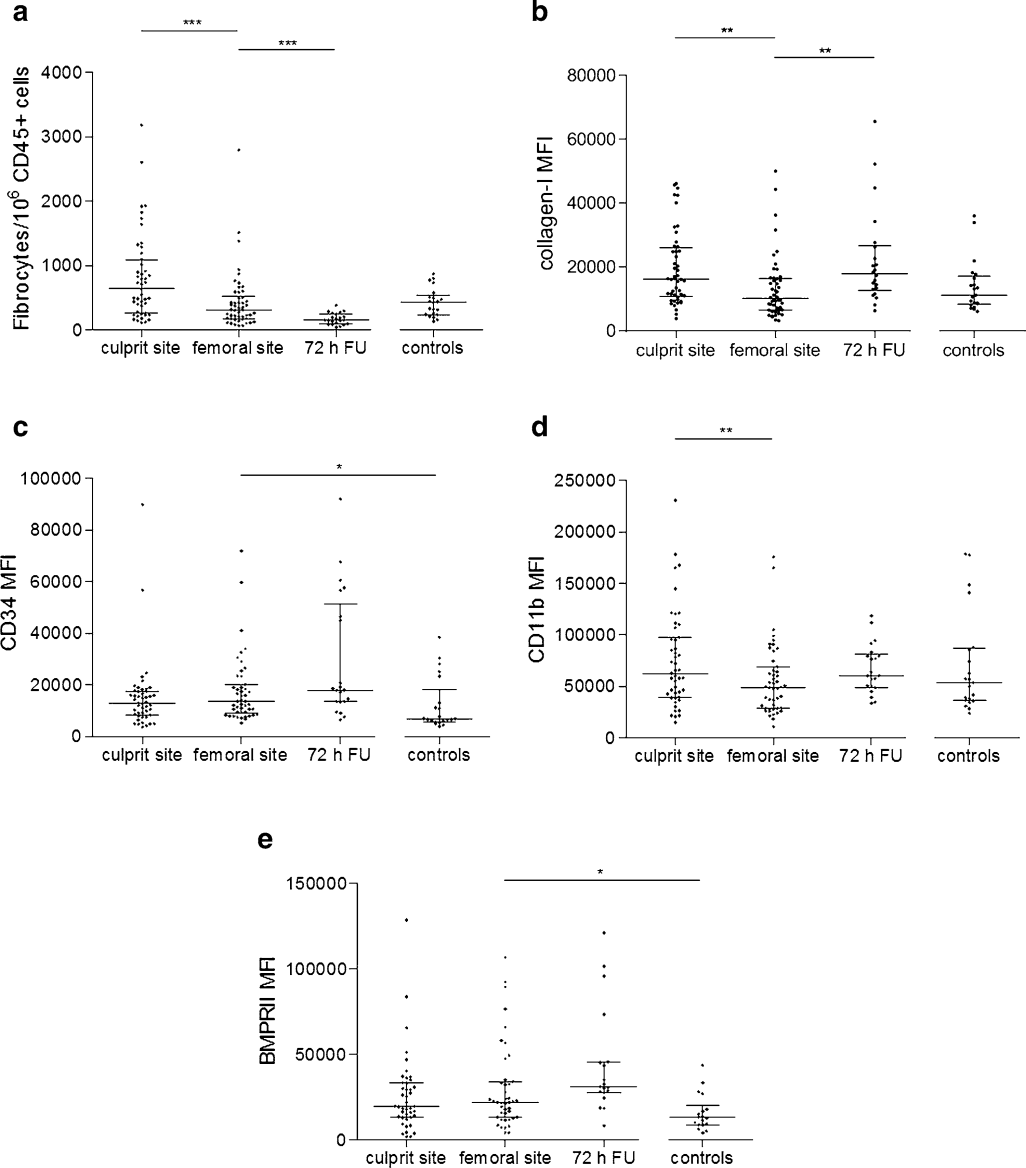
Circulating fibrocytes in ST-segment elevation myocardial infarction. Flow cytometry was used to analyze the frequency and activation of fibrocytes. Fibrocytes were identified by co-expression of CD45, CD34 and collagen-I (see Supplemental Fig. S2). Data are expressed as **a** number of fibrocytes/10^6^ CD45^+^ cells (baseline *n* = 50, 72 h FU *n* = 21, healthy *n* = 21) or mean fluorescence intensity (MFI) of **b** collagen-I (baseline *n* = 50, 72 h FU *n* = 21, healthy *n* = 21); **c** CD34 (baseline *n* = 50, 72 h FU *n* = 21, healthy *n* = 21); **d** CD11b (baseline *n* = 49, 72 h FU *n* = 21, healthy *n* = 21); **e** Bone morphogenic protein receptor II (BMPRII, baseline *n* = 43, 72 h FU *n* = 19, healthy *n* = 18). Respective n, numerical MFI values and statistical analyses are provided in Supplemental Tables 2, 3, and 4. Lines indicate median and interquartile range. Significance was determined by paired Wilcoxon signed-rank and unpaired Mann–Whitney *U* test. 72 h FU, follow-up at 72 h; *p < 0.05, **p < 0.01, ***p < 0.001

**Fig. 5:**
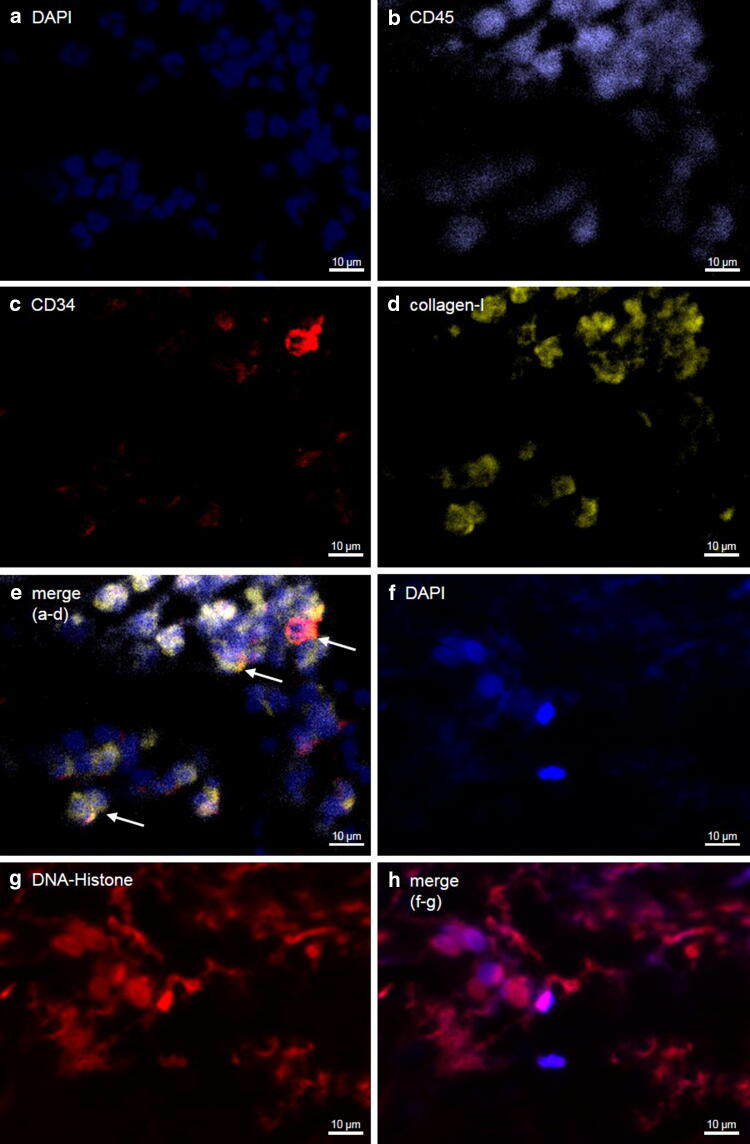
Fibrocytes and neutrophil extracellular traps in coronary culprit site thrombi. Thrombi were stained for **a** DAPI (blue); **b** CD45 (purple); **c** CD34 (red) and **d** collagen-I (yellow). **e** The merged image of sections **a**–**d**. White arrows mark fibrocytes. **f** For detection of NETs, thrombi were stained for **f** DAPI (blue) and **g** DNA-Histone (red). **h** The merged image for sections **f**-**g**

Circulating fibrocytes were significantly increased at the culprit site compared to the femoral site (Fig. 4a). At the culprit site, fibrocytes expressed increased levels of collagen-I (Fig. 4b), while CD34 expression was unchanged (Fig. 4c). However, CD11b expression was increased at the culprit site (Fig. 4d). Expression of BMPRII was not different between the culprit site and the femoral site (Fig. 4e). No correlations were found between symptom to balloon time, fibrocyte frequency or expression of surface markers (data not shown). By immunofluorescent staining, we detected CD45^+^CD34^+^collagen-I^+^ cells in particulate coronary thrombi, confirming the presence of fibrocytes (Fig. 5a–e). In line with previous results [38], we found NETs within thrombi (Fig. 5f–h).

We analyzed fibrocyte frequency and function 72 h after pPCI. Compared to baseline, we observed a decline of the number of circulating fibrocytes at 72 h (Fig. 4a). This was accompanied by increased collagen-I (Fig. 4b) but unchanged CD34, CD11b and BMPRII expression (Fig. 4c–e). BMPRII and collagen-I expressions of fibrocytes were positively correlated with each other (culprit site *n* = 43, *r*_s_ = 0.441, *p* < 0.01; femoral *n* = 43, *r*_s_ = 0.513, *p* < 0.001; 72 h *n* = 19, *r*_s_ = 0.802, *p* < 0.0001).

### Increased NET burden at the culprit site is associated with local fibrocyte activation

Since NETs were reported to promote fibroblast function [8], and based on our in vitro findings, we searched for a potential link between NET burden and fibrocyte function in patients. We observed a positive correlation between dsDNA at the culprit site and CD34 expression of culprit site fibrocytes (*n* = 47, *r*_s_ = 0.321, *p* < 0.05). Furthermore, a positive correlation was found between citH3 and CD34 expression (*n* = 47, *r*_s_ = 0.377, *p* < 0.01). A positive correlation was observed between dsDNA at the culprit site and BMPRII expression of culprit site fibrocytes (*n* = 41, *r*_s_ = 0.360, *p* < 0.05).

### Fibrocytes are present in the heart after myocardial infarction

**Fig. 6:**
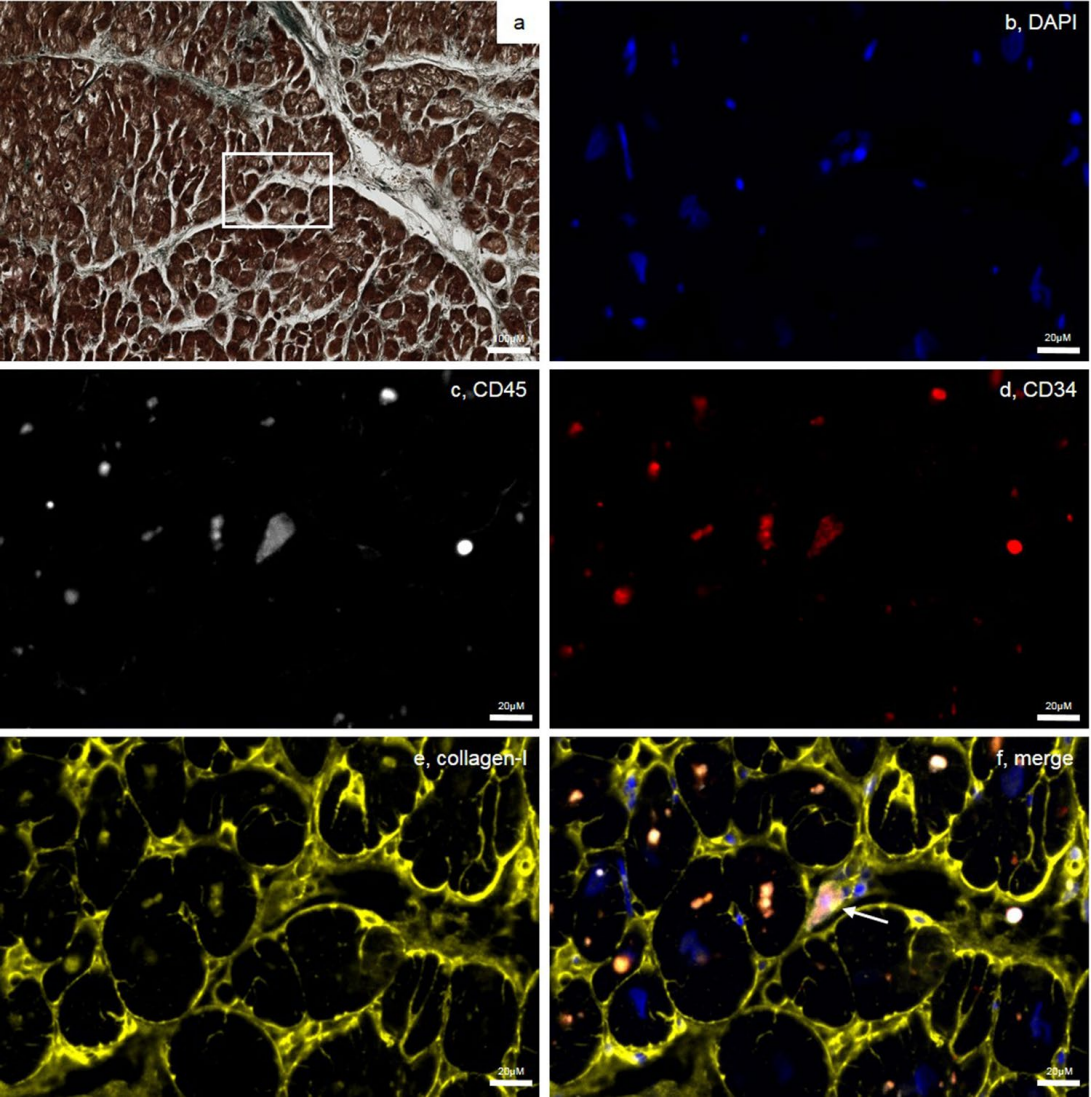
Fibrocytes in myocardium after myocardial infarction. Exemplary staining of tissue specimens from patients dying from STEMI using **a** a modified Trichrome stain and immunofluorescence. The white box indicates an area shown with immunofluorescence in **b**–**f**. Specimens were stained for **b** DAPI (blue); **c** CD45 (white); **d** CD34 (red) and **e** collagen-I (yellow). **f** represents the merged image. White arrow marks a fibrocyte

Fibrocytes were detected mostly in the transition zone between viable myocardium and scar of patients dying from acute STEMI (*n* = 4, 2.7 ± 0.6% of cells; Fig. 6). By contrast, few fibrocytes were detected in myocardial sections of normal hearts (*n* = 3, 0.9 ± 0.2%, *p* = 0.007 for STEMI vs. healthy; Supplemental Fig. S7).

Fibrocyte adhesion and migration are mediated via CD11b, with intracellular adhesion molecule 1 (ICAM-1) being an important ligand of CD11b [11, 50]. ICAM-1 has been shown to be upregulated in myocardium after ischemia [31, 47, 48]. Both ICAM-1 and CD11b were expressed in the transition zone after STEMI (Supplemental Fig. S8a, b).

### NET burden and fibrocyte activation at the culprit site are correlated with left ventricular dysfunction

**Fig. 7:**
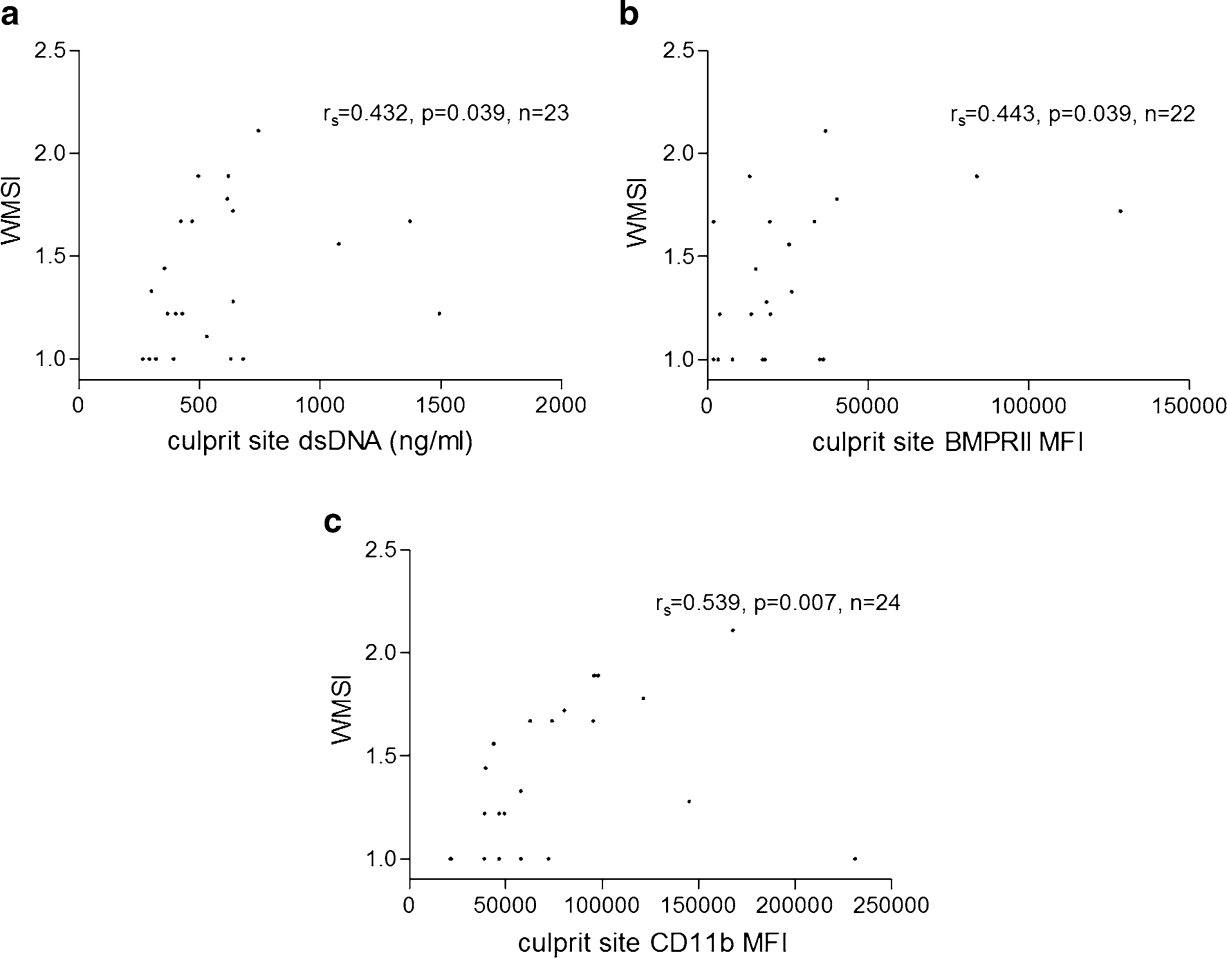
NETs marker and echocardiographic Wall Motion Score Index (WMSI) at follow-up (24 ± 8 months). **a** dsDNA at the culprit site and WMSI (*n* = 23); **b** WMSI and bone morphogenic protein receptor II (BMPRII) expression on culprit site fibrocytes (*n* = 22); **c** WMSI and CD11b expression on culprit site fibrocytes (*n* = 24). Correlation coefficients were calculated by Spearman’s rank correlation

In a subset of patients, we obtained transthoracic echocardiographic measurements at long-term follow-up. dsDNA at the culprit site at the time of pPCI was positively correlated with WMSI at follow-up (Fig. 7a). Fibrocyte numbers at the culprit site at baseline were not correlated with left ventricular function or WMSI at follow-up (data not shown). However, BMPRII (Fig. 7b) and CD11b (Fig. 7c) expressions on culprit site fibrocytes were positively correlated with WMSI.

## Discussion

**Fig. 8:**
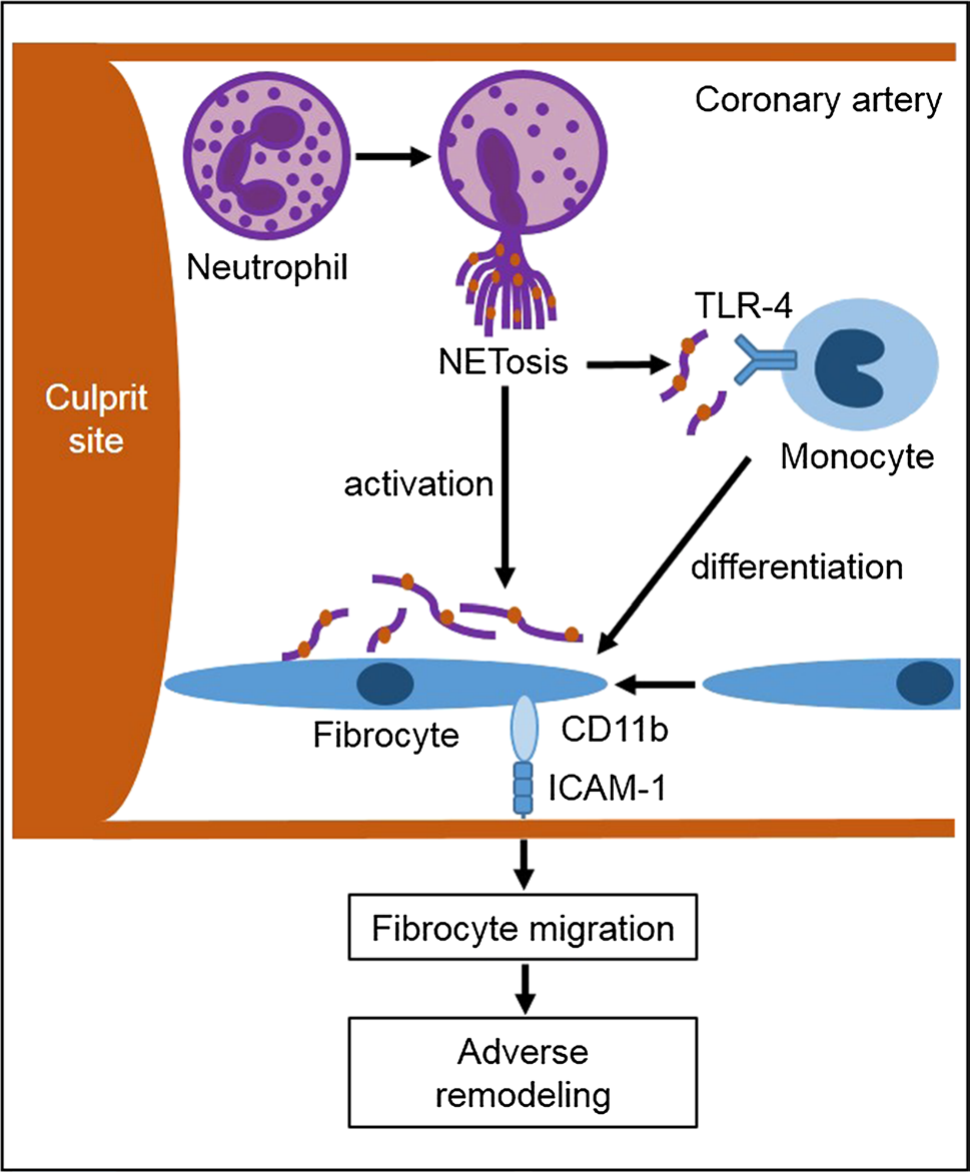
Graphical summary of the main findings. At the culprit site during ST-segment elevation myocardial infarction (STEMI), neutrophils undergo neutrophil extracellular trap (NET) formation. NETs activate fibrocytes and promote the differentiation of monocytes into fibrocytes, which can be antagonized by deoxyribonuclease and toll-like receptor (TLR)-4 blockade. Activated fibrocytes, expressing CD11b, adhere to the endothelium via interaction with intercellular adhesion molecule 1 (ICAM-1) and migrate into the myocardium to contribute to adverse remodeling

The conceptual framework is summarized in Fig. 8. We confirm that NET markers are increased locally at the culprit site and correlate with enzymatic infarct size. As new findings, we show that NETs induce fibrocyte differentiation from monocytes and activate fibrocytes in vitro. In vivo, fibrocytes accumulate at the culprit site in STEMI and in the myocardial transition zone, presumably contributing to myocardial tissue remodeling and affecting left ventricular function at follow-up. We demonstrate increased CD11b and BMPRII expression on fibrocytes, correlating with culprit site NET burden.

dsDNA was highly increased at the culprit site compared with the femoral site, confirming previous data [38, 60]. Furthermore, we observed increased peripheral concentrations of dsDNA in STEMI patients compared to healthy controls, which is in keeping with previous literature [7, 58]. dsDNA is a non-specific marker of cell death [51] and was shown to positively correlate with CK-MB, an established marker of myocardial necrosis [38, 58]. These findings suggest that in STEMI, dsDNA is elevated in the systemic circulation, presumably due to necrotic cell death. citH3 is a specific NET marker, indicating a specific local role at the site of coronary occlusion. This interpretation is supported by the observation that neutrophils isolated from the culprit site vessel were more prone to undergo NETosis upon ex vivo stimulation compared to neutrophils from healthy controls [60].

It was previously shown that fibroblasts are stimulated by NETs [8] and we found that NETs induced the differentiation of monocytes into fibrocytes, which was attenuated by DNase. Spindle-shaped fibrocytes [50] have been implicated in fibrotic diseases of the heart and lung [27, 36, 54], in atherosclerosis and in MI [13, 43]. To elucidate molecular mechanisms responsible for NET-induced fibrocyte differentiation, we investigated whether TLR signaling was involved. TLR-4, which is expressed on monocytes [68], has been shown to induce NET formation [63]. Apart from lipopolysaccharide, TLR-4 recognizes a variety of danger-associated molecular patterns, including high mobility group box 1 (HMGB1) [65], which is a component and important inducer of NETs [40, 42]. Addition of an anti-TLR-4 blocking antibody exerted a similarly inhibitory effect as DNase treatment. With regard to fibrocyte activation, NETs led to a significant upregulation of collagen-I, BMPRII and CD34 on these cells. IL-6, a pro-inflammatory cytokine that was demonstrated to be increased at the culprit site in myocardial infarction [15, 62] and is also produced by fibrocytes [53], was also increased upon stimulation with NETs. Addition of DNase only incompletely inhibited this pro-inflammatory response. Based on this observation, we conclude that the structural integrity of NETs is not required for all NET-mediated effects and that extracellular chromatin from other sources may produce overlapping effects.

In parallel to NETs, fibrocytes accumulate at the culprit site and are present in coronary thrombi. Increased expression of collagen-I on culprit site fibrocytes compared to the femoral site suggests that they become locally activated and might contribute to tissue remodeling via deposition of extracellular matrix. Collagen-I expression on peripheral fibrocytes 72 h after STEMI was upregulated and suggests a sustained systemic activation.

Hematopoietic stem cells have been shown to migrate to myocardial tissue after injury [1]. CD34 is expressed on hematopoietic progenitor cells [30] and has been used as a marker for the identification of fibrocytes [5, 50]. The biological role of CD34 remains incompletely understood; yet it has been suggested that CD34 mediates cell adhesion [16]. We observed increased CD34 levels on fibrocytes in STEMI compared to control, indicating increased adhesiveness of these cells in patients. Pre-formed intracellular CD34 can be released upon cell activation via a protein kinase C-dependent pathway [12]. This might be mediated by NETs, which can equally be triggered by protein kinase C [56]. In accordance with this, we observed a positive correlation between NET surrogate markers dsDNA and citH3 and CD34 expression of fibrocytes.

CD11b is expressed on fibrocytes [5, 50] and appears to be crucial for migration to target organs. For example, CD11b deficiency was associated with a lack of fibrocyte homing in a model of renal fibrosis [55]. In our study, CD11b expression on fibrocytes was increased at the culprit site. This might facilitate migration and adhesion to coronary arterial endothelium and enable subsequent migration of fibrocytes into the myocardium. CD11b recognizes a variety of ligands, including neutrophil elastase and myeloperoxidase [52], both of which are major components of NETs [4] and are increased at the culprit site [38]. NETs might, therefore, contribute to the chemotactic gradient that leads to influx of leukocytes, including fibrocytes, to the ischemic area. Increased adhesiveness of fibrocytes via CD11b appears to be detrimental for heart function, as CD11b expression was positively correlated with WMSI, a measurement of left ventricular dysfunction, at long-term follow-up. In a canine model of myocardial infarction, treatment with anti-CD11b antibody was associated with reduced neutrophil accumulation [59] and potentially also reduced fibrocyte migration.

The frequency of peripheral fibrocytes was lower 72 h after STEMI compared to baseline. We thought that fibrocytes might migrate to the ischemic myocardium. As expected, we detected fibrocytes within the myocardium of patients with STEMI. Our findings point towards homing of fibrocytes to the myocardium during and after STEMI. These observations are corroborated by a mouse model of ischemia/reperfusion cardiomyopathy showing an increase of fibrocytes in the heart after ischemic challenge [21]. A study of coronary artery ligation in mice reported myofibroblasts in the infarcted area, with bone marrow-derived fibrocytes as a potential source [67]. An autopsy study of coronary heart disease patients identified an increased fibrocyte number in heart tissue compared with control, which was correlated with collagen volume fraction [34]. Furthermore, peripheral fibrocytes were decreased in acute myocardial infarction compared to stable angina and healthy controls [13], suggesting fibrocyte homing. However, we did not observe a significant difference in peripheral fibrocyte count between patients and controls.

Several pathways have been implicated in atherosclerosis, among them bone morphogenic protein (BMP) signaling [6]. BMP receptor type II (BMPRII) is expressed in a variety of tissues and cells, including fibroblasts [18], and significantly modulates BMP signaling [44]. Depending on ligand concentration, context and cell type, BMP signaling mediates multiple, often contrary effects on target cells [45]. BMPRII plays an important role in endothelial cell and vascular physiology [44]. Mutations of BMPRII lead to a hereditary form of pulmonary hypertension characterized by severe intimal proliferation and medial hypertrophy [32]. BMPRII deficiency results in endothelial dysfunction and inflammation, and BMPRII disappears in advanced atherosclerotic plaques [29]. On the other hand, heterozygous deletion of BMP-4, a major ligand of BMPRII, results in decreased infarct size in a murine model of transient ischemia/reperfusion [49]. In another study, BMP-4 induced collagen production of cardiac fibroblasts in vitro [61]. BMPRII and collagen-I expressions were upregulated on fibrocytes in STEMI. BMPRII expression during STEMI was positively correlated with increased WMSI at long-term follow-up. Our findings suggest that BMPRII on fibrocytes, via excess production of collagen-I, is associated with worse cardiac function.

Soluble NET markers were shown to be associated with adverse cardiac events [2]. The present study was not powered to assess clinical cardiovascular endpoints; however, both dsDNA and citH3 were positively correlated with enzymatic infarct size, and dsDNA positively correlated with WMSI at long-term follow-up. Thus, high NET burden not only mediates bigger infarct size [38], but impacts global cardiac function.

Currently, limited evidence exists on cardioprotective strategies for reducing myocardial damage in MI [23]. Thus, there is an urgent need for defining novel therapeutic targets. Although a causal relationship needs to be established, our data suggest that NETs might be important mediators of fibrotic remodeling after STEMI, possibly by stimulating fibrocytes.

## Limitations

This is a translational, hypothesis-generating study. Given the observational nature of our findings, causality cannot be inferred. citH3 has been extensively used for the identification of NETs both on the tissue level [33, 57, 69] and as a soluble surrogate marker [41, 66]. dsDNA has also been used to measure NET burden [2, 38, 66], although specificity is low [51]. Therefore, some of our results may be attributed to extracellular chromatin from sources other than NETosis.

Measurement of DNase as the natural counter-regulatory mechanism of nucleosome generation in STEMI was not performed because two different DNase types are relevant in vascular biology and would have been needed to be addressed [25]. In this manuscript, the role of DNase was limited to in vitro experiments. Cardiac MRT was not available in those patients in whom fibrocytes were analyzed. Therefore, cardiac dysfunction was assessed by echocardiography in a subset of 24 patients at long-term follow-up. Our study was not powered for assessment of outcome. Larger studies are required to establish causality.

## Electronic supplementary material

Below is the link to the electronic supplementary material.

Supplementary material 1 (DOCX 923 kb)

## Abbreviations

ACE: Angiotensin converting enzyme inhibitor
ARB: Angiotensin receptor blocker
ASA: Acetylsalicylic acid
AUC: Area under the curve
BMI: Body mass index
BMP: Bone morphogenic protein
BMPRII: Bone morphogenic protein receptor II
CAD: Coronary artery disease
CD: Cluster of differentiation
citH3: Citrullinated histone H3
CK-MB: Creatine-phosphokinase isoform MB
CRP: C-reactive protein
CX: Circumflex artery
DNase: Deoxyribonuclease
dsDNA: Double-stranded deoxyribonucleic acid
EDTA: Disodium ethylenediaminetetraacetate
EV-TF: Extracellular vesicle-associated tissue factor
FCS: Fetal calf serum
FU: Follow-up
FX: Factor X
HDL: High-density lipoprotein
IQR: Interquartile range
LAD: Left anterior descending artery
LDL: Low-density lipoprotein
LVEF: Left ventricular ejection fraction
MFI: Mean fluorescence intensity
MI: Myocardial infarction
NET: Neutrophil extracellular traps
PBMC: Peripheral blood mononuclear cells
PBS: Phosphate-buffered saline
pPCI: Primary percutaneous coronary intervention
RCA: Right coronary artery
SD: Standard deviation
STEMI: ST-segment elevation myocardial infarction
TIMI: Thrombolysis in myocardial infarction
TnT: Troponin T
VD: Vessel disease
WMSI: Wall Motion Score Index
