# Supplementary material for: Neutrophil extracellular traps and fibrocytes in ST-segment elevation myocardial infarction
Source: Basic Res Cardiol. 2019 Jul 16;114(5):33. doi: 10.1007/s00395-019-0740-3 (PMC6647191; doi:10.1007/s00395-019-0740-3)
Supplement: Supplementary file 1 — Supplementary material 1 (DOCX 923 kb) [file 395_2019_740_MOESM1_ESM.docx]

**Supplemental Material**

**Neutrophil extracellular traps and fibrocytes in ST-segment elevation myocardial infarction**

# **Supplemental Methods and Results**

**In vitro catheter aspiration assay and flow cytometry for neutrophil extracellular traps and fibrocyte activation.**

To exclude an influence of the catheter on neutrophils and NET formation during thrombus aspiration, we performed an *in vitro* catheter aspiration assay as previously described [2]. EDTA whole blood samples (n=3) were aspirated through thrombectomy catheters (Export, Medtronic). After discarding the first 1 ml of sample, blood was used for subsequent the experiment. NETotic neutrophils were quantified as previously described [1, 3], with minor modifications. Healthy control EDTA whole blood was mixed 3:10:5 in PBS buffer containing 15 nM EDTA, 1% BSA and 6% Hetastarch, respectively and incubated for 40 min at 37 °C to allow sedimentation of erythrocytes. Cells were collected by harvesting the supernatant and resuspended in PBS. Samples were centrifuged at 1500 rpm for 5 min, after which the cell pellet was resuspended in RPMI. NET formation was then induced using 0.5 µM ionomycin for 2 h at 37 °C. After stimulation, cells were fixed with 4% paraformaldehyde for 30 min, washed in PBS and then blocked using PBS + 2% bovine serum albumine (BSA). Then, a rabbit anti-citH3 antibody was added for 20 min at 21 °C in the dark. Cells were washed and incubated with a secondary anti-rabbit and anti-CD66b antibodies for 20 min. After three washing steps, cells were resuspended in PBS containing propidium iodide. Using flow cytometry, neutrophils were identified as propidium iodide- and CD66b-positive cells. NETotic neutrophils were gated using citH3 positivity and computed as percentage of total neutrophils. We found no difference in the percentage of NETotic neutrophils between samples with our without prior catheter aspiration (Supplemental Fig. S3a), irrespective of *ex vivo* stimulation with ionomycin.

A fraction of whole blood subjected to catheter aspiration and respective control was processed for flow cytometry to analyze fibrocyte activation, as described in the Main Methods. We did not observe activation of fibrocytes by catheter aspiration as measured by expression levels of collagen-I, BMPRII, CD34 and CD11b (Supplemental Fig. S3b).

**Immunohistochemistry**

For measurement of culprit site thrombus NET burden, we employed immunohistochemistry as previously described [2]. Thrombi were incubated using a primary antibody against DNA-Histone H1 for 1 h at 21 °C. Furthermore, infarcted myocardium was stained using primary antibodies against ICAM-1 and CD11b for 1 h at 21 °C. Samples were then processed using a Histostain-SP IHC Kit according to manufacturer’s instruction. Images were acquired with an Axio Observer Z1 fluorescence microscope. Analysis of images was performed using TissueQuest software (TissueGnostics, version 4.01.0128).

# **Supplemental References**

1. Gavillet M, Martinod K, Renella R, Harris C, Shapiro NI, Wagner DD, Williams DA (2015) Flow cytometric assay for direct quantification of neutrophil extracellular traps in blood samples. Am J Hematol 90:1155-1158 doi:10.1002/ajh.24185

2. Mangold A, Alias S, Scherz T, Hofbauer T, Jakowitsch J, Panzenbock A, Simon D, Laimer D, Bangert C, Kammerlander A, Mascherbauer J, Winter MP, Distelmaier K, Adlbrecht C, Preissner KT, Lang IM (2015) Coronary neutrophil extracellular trap burden and deoxyribonuclease activity in ST-elevation acute coronary syndrome are predictors of ST-segment resolution and infarct size. Circulation Research 116:1182-1192 doi:10.1161/CIRCRESAHA.116.304944

3. Martinod K, Witsch T, Erpenbeck L, Savchenko A, Hayashi H, Cherpokova D, Gallant M, Mauler M, Cifuni SM, Wagner DD (2017) Peptidylarginine deiminase 4 promotes age-related organ fibrosis. J Exp Med 214:439-458 doi:10.1084/jem.20160530

# **Supplemental Figure S1**


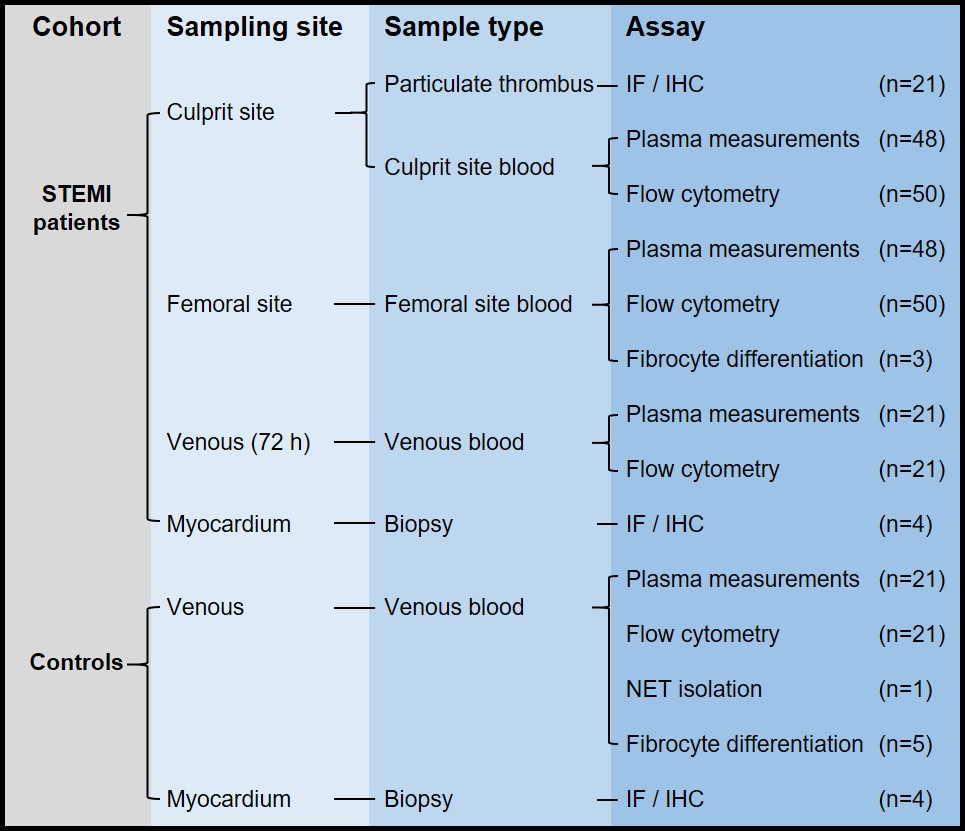


**Schematic representation of sampling strategy and subsequent experimental procedures**. *IF* immunofluorescence, *IHC* immunohistochemistry, *NET* Neutrophil extracellular trap, *STEMI* ST-segment elevation myocardial infarction

# **Supplemental Figure S2**


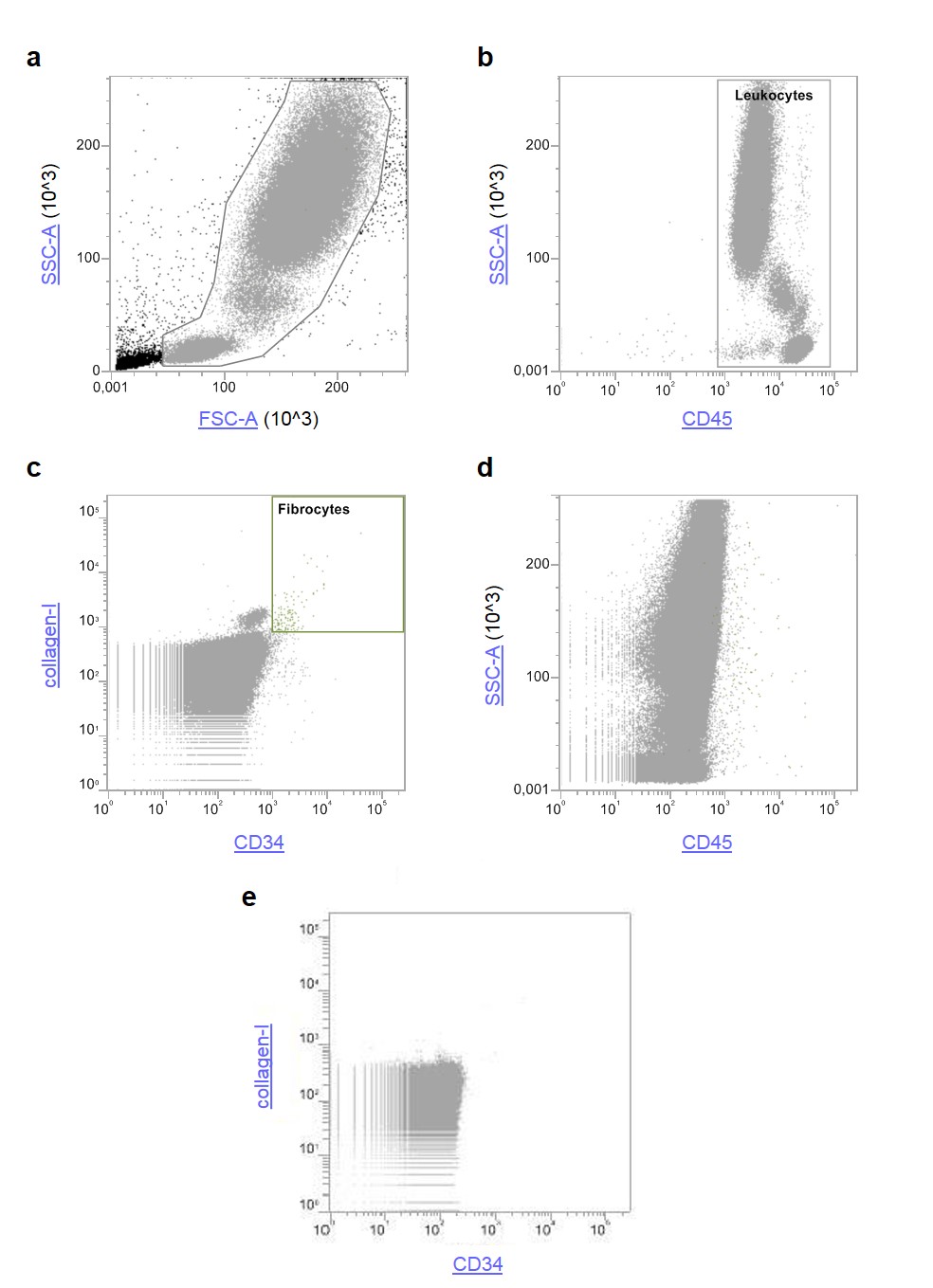


**Exemplary flow cytometric gating strategy of circulating fibrocytes**. **a**, Based on forward scatter (FSC) and side scatter (SSC), cells are separated from debris. **b**, Leukocytes are identified by positivity for CD45. **c**, Fibrocytes are then identified based on positivity for collagen-I and CD34. **d-e**, unstained control samples

# **Supplemental Figure S3**

**
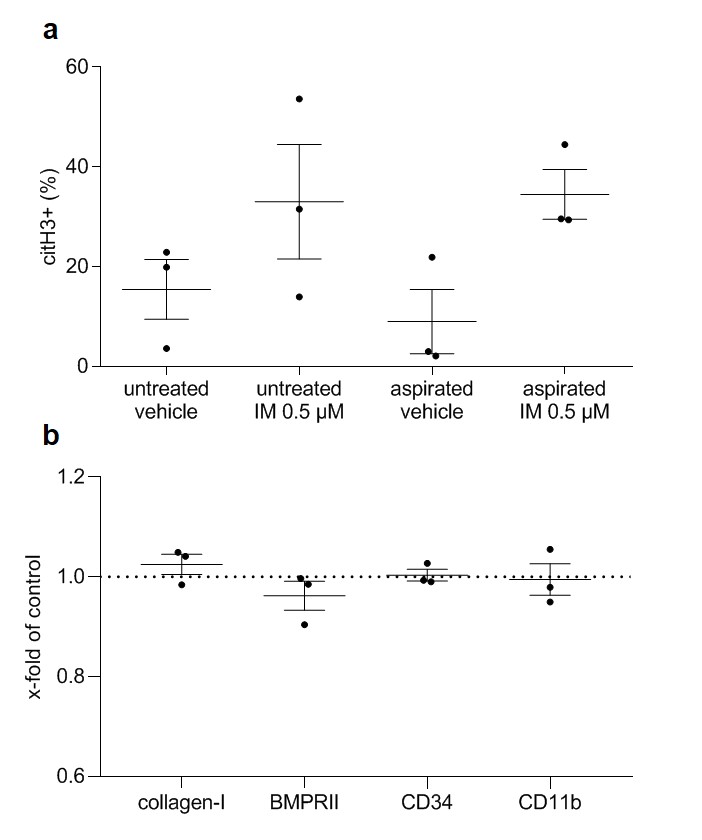
**

**Catheter aspiration and NET formation and fibrocyte activation. a,** neutrophils were stimulated using 0.5 µM ionomycin or vehicle to assess the influence of catheter aspiration on NETosis (n=3). Cells were then analyzed using flow cytometry. Data are given as percentage of citH3-positive, i.e. NETotic neutrophils. **b**, after catheter aspiration, whole blood was stained for fibrocyte markers. Data are given as x-fold of non-aspirated control

# **Supplemental Figure S4**


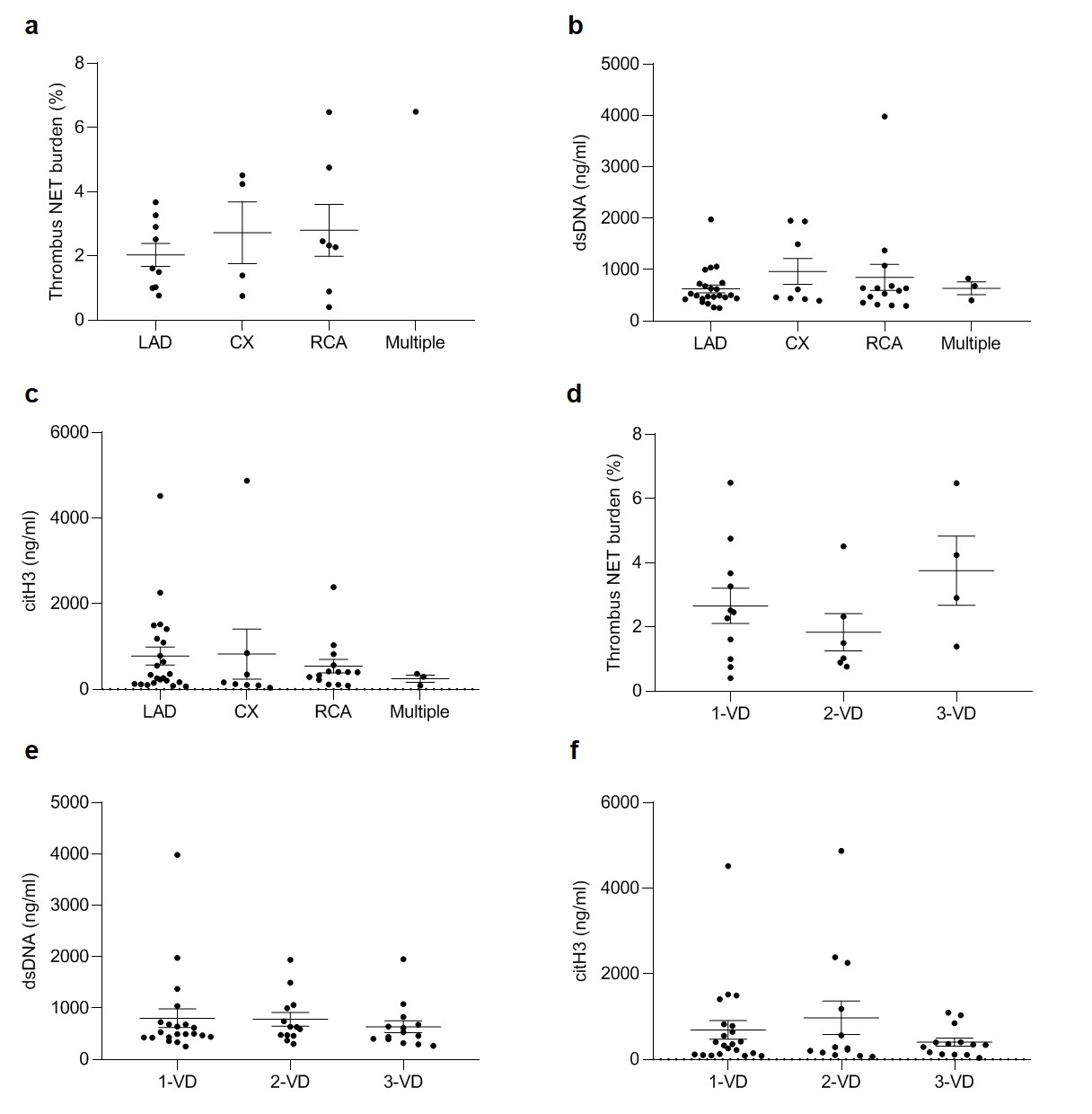


**Comparison of NET burden in culprit site thrombi and plasma between locations of culprit site and extent of coronary artery disease**

# **Supplemental Figure S5**

**
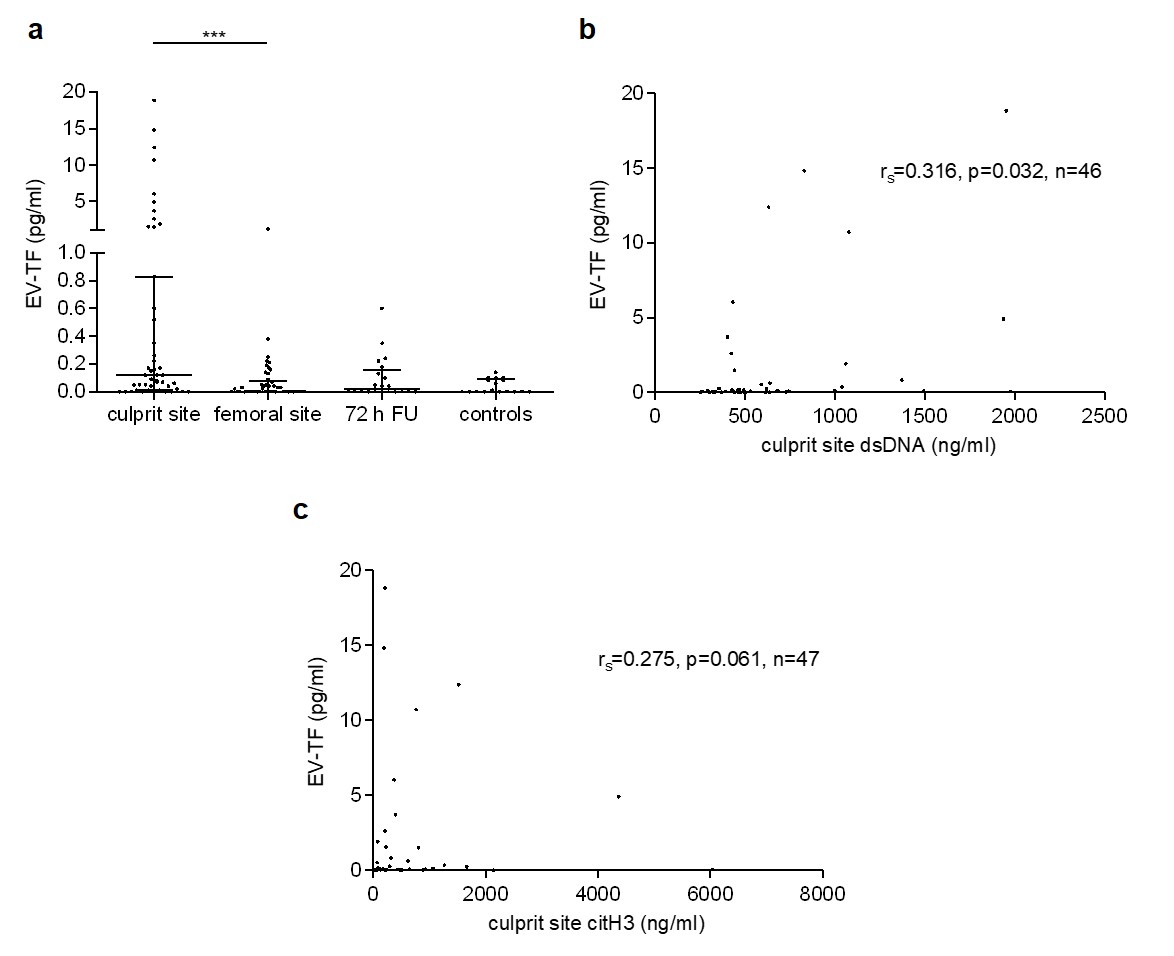
**

**Measurement of extracellular vesicle-associated tissue factor (EV-TF) in patients with ST-segment elevation myocardial infarction (STEMI) and healthy controls, and correlation with neutrophil extracellular traps (NETs) surrogate markers. a,** EV-TF activity was measured using Pefachrome FXa. Data are expressed as median and interquartile range. **b**, correlation of EV-TF activity with culprit site double-stranded (ds)DNA. **c**, correlation of EV-TF activity with culprit site citrullinated histone H3 (citH3). Correlation coefficients were calculated by Spearman’s rank correlation. * p<0.001

# **Supplemental Figure S6**


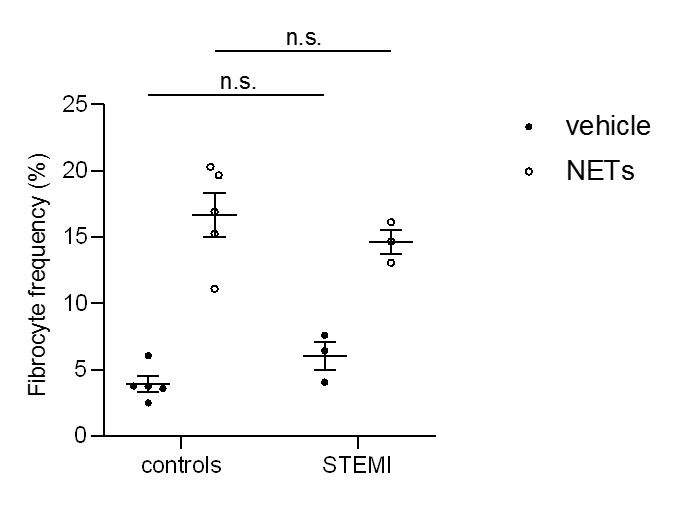


**Differentiation of monocytes into fibrocytes in controls and STEMI patients.** Isolated peripheral blood mononuclear cells containing monocytes from healthy controls (n=5) and STEMI patients (n=3) were stimulated with vehicle control or 500 ng/ml isolated NETs. Data are given as mean ± standard error of the mean. n.s. not significant

# **Supplemental Figure S7**

**
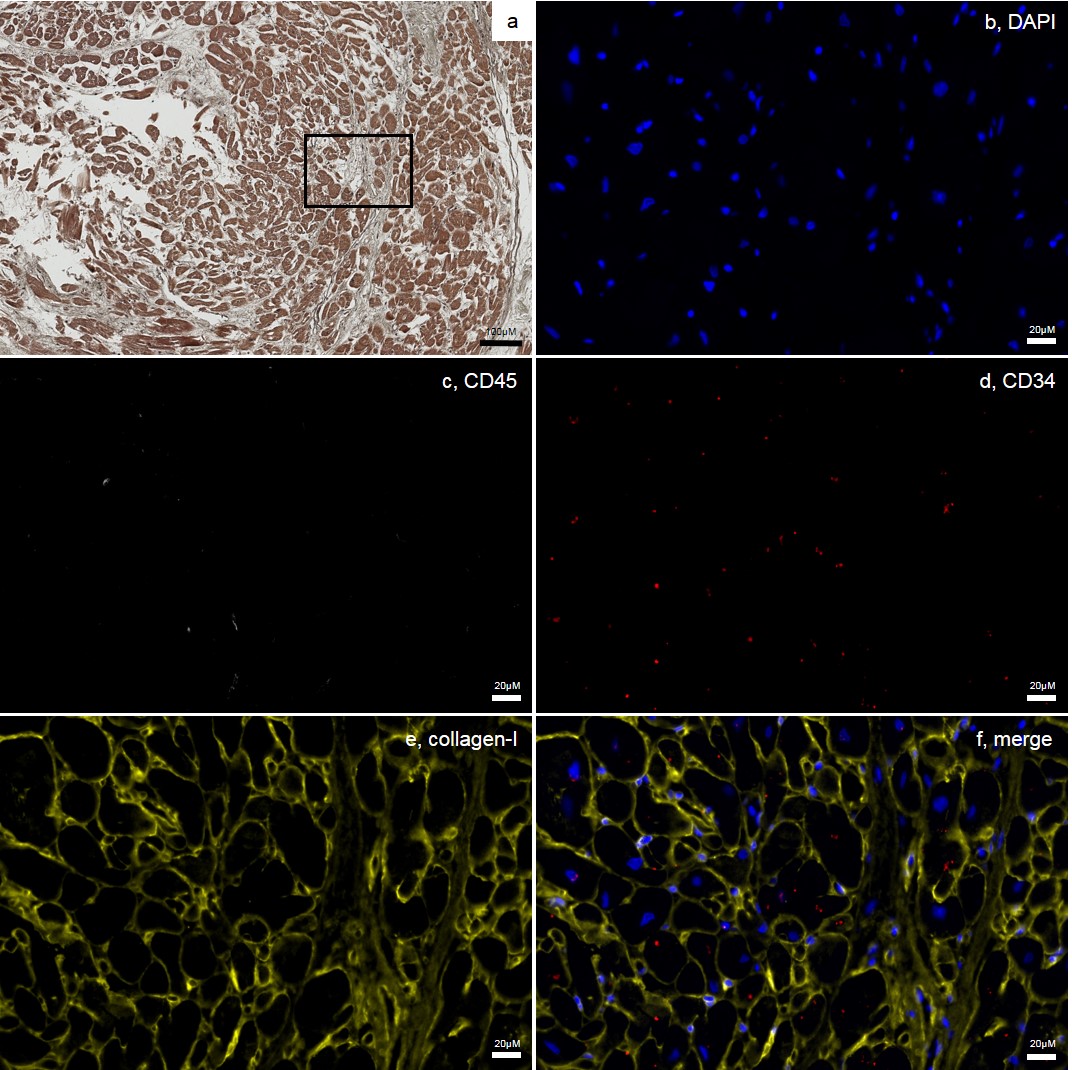
**

**Immunofluorescent staining of healthy myocardium.** Tissue specimens from patients suffering from ischemic heart disease were stained using **a**, modified Trichrome and immunofluorescence. White box indicates area shown for immunofluorescent staining. Specimens were stained for **b**, DAPI (blue); **c**, CD45 (white); **d**, CD34 (red) and **e**, collagen-I (yellow). **f** represents the merged image

# **Supplemental Figure S8**

**
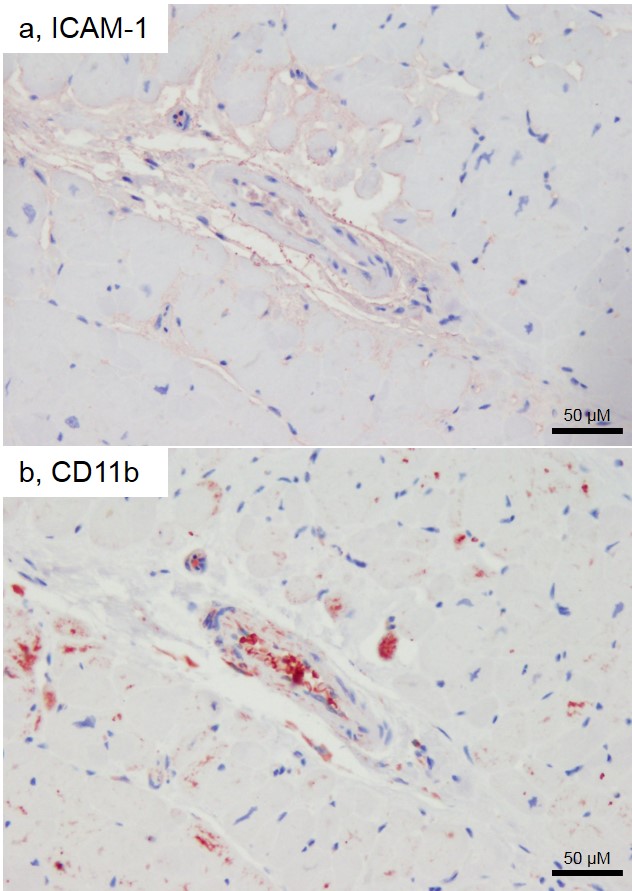
**

**CD11b and ICAM-1 expression of myocardium after STEMI.**  Using immunohistochemistry, tissue specimens from patients suffering from ischemic heart disease were stained for **a**, intercellular adhesion molecule (ICAM)-1 and **b**, CD11b

# **Supplemental Table 1 – Major Resources**

| Reagent | Company | Catalog # | Dilution |
| --- | --- | --- | --- |
|  |  |  |  |
| Soluble markers |  |  |  |
| Quant-iT PicoGreen kit | ThermoFisher | P7589 |  |
| Cell Death Detection ELISA Plus kit | Roche | 11774425001 |  |
| citrullinated histone H3 | Cayman | 17926 |  |
| rabbit polyclonal anti-human citrullinated histone H3 | Abcam | ab5103 | 1:2000 |
| goat anti-rabbit HRP conjugate | BioRad | 170-6515 | 1:5000 |
| BM Blue POD Substrate | Roche | 11484281001 |  |
| mouse anti-human tissue factor antibody | BD Biosciences | 550252 | 500 µg/ml |
| control mouse IgG antibody | Sigma Aldrich | I5381 | 500 µg/ml |
| Factor VIIa | Coachrom | HF7a |  |
| Factor X | Coachrom | HF10 |  |
| Pefachrome FXa | Pentapharm | 085-17 |  |
|  |  |  |  |
| Cell Culture |  |  |  |
| Lymphocyte Separation Medium | PromoCell | C-44010 |  |
| Dextran T500 | Pharmacosmos | 551005009006 |  |
| Phorbol myristate acetate | Sigma-Aldrich | P1585 |  |
| MEM non-essential amino acids | Sigma-Aldrich | M7145 |  |
| BD GolgiPlug Protein Transport Inhibitor | Becton Dickinson | 555029 |  |
| Pulmozyme (human Dornase alfa) | La Roche Ltd. | N/A |  |
| anti-TLR-4 blocking antibody | Invivogen | pab-hstlr4 |  |
| Trypsin EDTA | Lonza | BE17-161E |  |
|  |  |  |  |
| Flow cytometry |  |  |  |
| mouse IgG1 anti-human collagen-I (FITC) antibody | Merck-Millipore | FCMAB412F | 1:200 |
| mouse IgG1 anti-human CD34 (PerCP/Cy5.5) antibody | Biolegend | 343522 | 1:40 |
| mouse IgG1 anti-human CD45 (APC-Cy7) antibody | Biolegend | 304014 | 1:40 |
| mouse IgG1 anti-human CD11b (PE-Cy7) antibody | Biolegend | 301322 | 1:40 |
| goat polyclonal anti-human BMPRII (unconjugated) antibody | R&D Systems | AF811 | 1:40 |
| donkey polyclonal anti-goat IgG (PE) 2^nd^ antibody | R&D Systems | F0107 | 1:40 |
| rat IgG1 anti-human IL-6 (APC) antibody | Biolegend | 501112 | 1:40 |
| BD FACS Lysis Solution | Becton Dickinson | 349202 |  |
| FIX & PERM fixation medium | Nordic MUbio | GAS-002A-1 |  |
| FIX & PERM permeabilization medium | Nordic MUbio | GAS-002B-1 |  |
| Heta Starch | Braun | L6511 |  |
| EDTA | Sigma-Aldrich | E6758 |  |
| Bovine serum antigen (BSA) | MP Biomedicals | 160069 |  |
| Ionomycin | Sigma-Aldrich | I3909 |  |
| Paraformaldehyde | Sigma-Aldrich | D8537 |  |
| rabbit unconjugated anti-human citH3 | Abcam | ab5103 | 1:180 |
| goat Alexa Fluor 647 anti-rabbit | Invitrogen | A21246 | 1:10000 |
| mouse Pacific Blue anti-human CD66b | Biolegend | 305112 | 1:40 |
| Propidium iodide (PI) | BD Biosciences | 556547 | 1:50 |
|  |  |  |  |
| Immunofluorescence and immunohistochemistry |  |  |  |
| rat IgG2a anti-human CD34 primary antibody | ThermoFisher | MA1-22646 | 1:30 |
| donkey polyclonal anti-rat IgG DyLight 550 2^nd^ antibody | ThermoFisher | SA5-10027 | 1:500 |
| mouse IgG1 anti-human CD45 primary antibody | Abcam | ab8216 | 1:250 |
| mouse IgG2a anti-human DNA-Histone H1 antibody | Merck | MAB3864 | 1:500 |
| donkey polyclonal anti-mouse IgG DyLight 755 2^nd^ antibody | ThermoFisher | SA5-10171 | 1:250 |
| rabbit polyclonal anti-human collagen-I primary antibody | Abcam | ab34710 | 1:200 |
| donkey polyclonal anti-rabbit IgG DyLight 650 2^nd^ antibody | Abcam | ab96922 | 1:500 |
| 4′,6-Diamidin-2-phenylindol | Sigma | D9542 | 1:500 |
| mouse monoclonal anti-human ICAM-1 antibody | Abcam | ab2213 | 1:100 |
| rabbit monoclonal anti-human CD11b antibody | Abcam | ab133357 | 1:300 |
| Histostain-SP IHC Kit, AEC, broad spectrum | Invitrogen | 959943 |  |

# **Supplemental Table 2 – culprit site versus femoral**

Characterization of fibrocytes at the culprit site and at the femoral site. Numerical values of mean fluorescence intensity (MFI) of surface markers on fibrocytes refer to data illustrated in Figure 4. Respective n are given in each row. Data are given as median [interquartile range, IQR] frequency / 10^6^ CD45+ cells or MFI. Adjusted (adj.) p values of paired t-tests were computed based on Bonferroni-Holm method.

|  | n | unit | culprit site | femoral | p-value | p value adj. | Figure |
| --- | --- | --- | --- | --- | --- | --- | --- |
| fibrocyte count | 50 | median [IQR]  /10^6^ CD45+ cells | 677 [276-1298] | 311 [176-523] | 0.000023 | 0.000115 | 4a |
| collagen-I | 50 | median [IQR] MFI | 16313 [11062-25461] | 9824 [6610-16474] | 0.000466 | 0.001864 | 4b |
| CD34 | 50 | median [IQR] MFI | 13290 [8527-18041] | 13606 [8921-19883] | 0.447 | 0.894 | 4c |
| CD11b | 49 | median [IQR] MFI | 60056 [39219-96828] | 49311 [28801-71942] | 0.000489 | 0.001467 | 4d |
| BMPRII | 43 | median [IQR] MFI | 19599 [13229-33400] | 22106 [13142-34125] | 0.506612 | 0.506612 | 4e |

# **Supplemental Table 3 – femoral versus 72 h**

Characterization of fibrocytes at the femoral site and 72 h after STEMI. Numerical values of mean fluorescence intensity (MFI) of surface markers on fibrocytes refer to data illustrated in Figure 4. Respective n are given in each row. Data are given as median [interquartile range, IQR] frequency / 10^6^ CD45+ cells or MFI. Adjusted (adj.) p values of unpaired t-tests were computed based on Bonferroni-Holm method.

|  | n | unit | femoral | 72h follow-up | p value | p value adj. | Figure |
| --- | --- | --- | --- | --- | --- | --- | --- |
| fibrocyte count | 21 | median [IQR]  /10^6^ CD45+ cells | 311 [176-523] | 153 [102-252] | 0.000100 | 0.0005 | 4a |
| collagen-I | 21 | median [IQR] MFI | 9706 [7038-16238] | 17512 [12421-27127] | 0.001570 | 0.00628 | 4b |
| CD34 | 21 | median [IQR] MFI | 14229 [11278-18704] | 18020 [13514-51571] | 0.179 | 0.179 | 4c |
| CD11b | 21 | median [IQR] MFI | 48206 [28205-62157] | 60498 [48834-82091] | 0.033340 | 0.06668 | 4d |
| BMPRII | 19 | median [IQR] MFI | 22294 [17973-34125] | 31149 [27722-45724] | 0.024945 | 0.074835 | 4e |

**Supplemental Table 4 – femoral STEMI versus control**

Characterization of fibrocytes at the femoral site in STEMI patients and in healthy controls. Numerical values of mean fluorescence intensity (MFI) of surface markers on fibrocytes refer to data illustrated in Figure 4. Respective n are given in each row. Data are given as median [interquartile range, IQR] frequency / 10^6^ CD45+ cells or MFI. Adjusted (adj.) p values were computed based on Bonferroni-Holm method.

|  | n | unit | STEMI femoral | control | p value | p value adj. | Figure |
| --- | --- | --- | --- | --- | --- | --- | --- |
| fibrocyte count | 50 / 21 | median [IQR]/  10^6^ CD45+ cells | 311 [176-523] | 428 [235-540] | 0.236299 | 1.0 | 4a |
| collagen-I | 50 / 21 | median [IQR] MFI | 9824 [6610-16474] | 11309 [8450-17229] | 0.331992 | 0.331992 | 4b |
| CD34 | 50 / 21 | median [IQR] MFI | 13606 [8921-19883] | 6950 [5875-18275] | 0.005977 | 0.023908 | 4c |
| CD11b | 49 / 21 | median [IQR] MFI | 49311 [28801-71942] | 53585 [36355-86712] | 0.320592 | 0.641184 | 4d |
| BMPR-II | 43 / 18 | median [IQR] MFI | 22106 [13142-34125] | 13099 [8944-20231] | 0.009863 | 0.029589 | 4e |
